# The burden of perioperative hypertension/hypotension: A systematic review

**DOI:** 10.1371/journal.pone.0263737

**Published:** 2022-02-09

**Authors:** Irene Lizano-Díez, Stephen Poteet, Adrià Burniol-Garcia, Mónica Cerezales

**Affiliations:** 1 Ferrer, Barcelona, Spain; 2 Axentiva Solutions SL, Barcelona, Spain; Cleveland Clinic, UNITED STATES

## Abstract

**Study objective:**

Our goal is to review the outcomes of acute hypertensive/hypotensive episodes from articles published in the past 10 years that assessed the short- and long-term impact of acute hypertensive/hypotensive episodes in the perioperative setting.

**Methods:**

We conducted a systematic peer review based upon PROSPERO and Cochrane Handbook protocols. The following study characteristics were collected: study type, author, year, population, sample size, their definition of acute hypertension, hypotension or other measures, and outcomes (probabilities, odds ratio, hazard ratio, and relative risk) and the p-values; and they were classified according to the type of surgery (cardiac and non-cardiac).

**Results:**

A total of 3,680 articles were identified, and 66 articles fulfilled the criteria for data extraction. For the perioperative setting, the number of articles varies by outcome: 20 mortality, 16 renal outcomes, 6 stroke, 7 delirium and 34 other outcomes. Hypotension was reported to be associated with mortality (OR 1.02–20.826) as well as changes from the patient’s baseline blood pressure (BP) (OR 1.02–1.36); hypotension also had a role in the development of acute kidney injury (AKI) (OR 1.03–14.11). Postsurgical delirium was found in relation with BP lability (OR 1.018–1.038) and intra- and postsurgical hypotension (OR 1.05–1.22), and hypertension (OR 1.44–2.34). Increased OR (37.67) of intracranial hemorrhage was associated to postsurgical systolic BP >130 mmHg. There was a wide range of additional diverse outcomes related to hypo-, hypertension and BP lability.

**Conclusions:**

The perioperative management of BP influences short- and long-term effects of surgical procedures in cardiac and non-cardiac interventions; these findings support the burden of BP fluctuations in this setting.

## Introduction

Perioperative blood pressure (BP) variability, hypertension (HTN) and hypotension (HPT) have all been associated with hemodynamic instability and poor clinical outcomes [1]. Optimal pharmacologic control of BP requires intravenous (IV) agents that are easy to prepare and administer and that have rapid onset and offset of action that allows a predictable effect and easy dose-titration to properly fine-tune the BP of the patient, among other requirements [1].

Treatment choices for acute HTN depend on several factors in addition to BP measurement. These include evidence of end-organ damage (e.g., cerebral, cardiac, vascular, renal) presence of comorbidities (e.g., aortic dissection, acute myocardial infarction (AMI), bleeding) and ability to ingest and absorb oral medicines [2]. Examples of such clinical circumstances include perioperative HTN, in which rapid control of BP is essential to limit or prevent end-organ injury.

Both HTN and HPT in perioperative settings or acute HTN may result in a high economic burden for healthcare systems [3–5] due to perioperative complications requiring prolonged hospitalization.

Even though there are several international reference guidelines that account for the importance of management of perioperative BP [6–8], at present there are no universally accepted preoperative BP thresholds, as BP targets need to consider patient baseline BP, surgery type, and risk of short-term complications [9]. Furthermore, there is a clear gap in the knowledge of short- and long-term implications of acute hypo- and hypertensive perioperative episodes. Thus, a comprehensive, systematic review would have important and broad implications, particularly due to anecdotal evidence that suggests substantial underutilization of antihypertensive agents by clinical area.

The objective of this research is to systematically review the available evidence on critical outcomes potentially associated with hypertensive/hypotensive episodes (e.g., mortality, stroke, AMI, acute kidney injury (AKI), and others) in the perioperative setting for cardiac and non-cardiac surgeries, and to compare these findings to serve as a guide for clinical decision making, health policy and future research.

## Methods

### Study design

**Fig 1:**
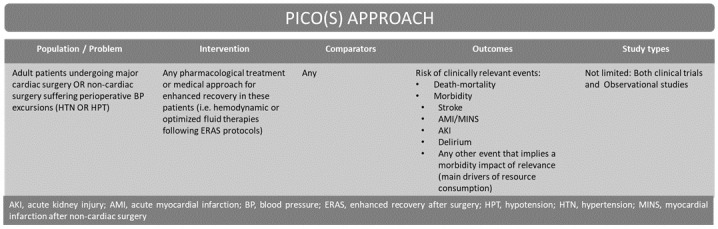
PICO(S) search strategy.

A systematic review of the literature was conducted in order to identify studies that analyzed the implications of HTN and HPT in the perioperative setting. The study design was formulated based upon PROSPERO and Cochrane Handbook protocols (Fig 1 and S1 Checklist and S1 File). The research protocol was registered in Open Science Framework (https://osf.io/vgjmu).

### Inclusion and exclusion criteria

Studies were included based on the following criteria:

1. Original research articles from January 1, 2010, to December 31, 2020. The last ten calendar years to collect the latest evidence published.
2. Population 18 years of age or older.
3. Outcomes of acute hypertension/hypotension episodes in the perioperative settings.

Studies were excluded based on:

1. Key terms were not included in either the title or abstract (perioperative, intensive care, HTN, HPT, and BP).
2. Title or abstract included the following terms: pulmonary, intra-abdominal compartment syndrome, children, pediatric, infant, or pregnancy.
3. Non-English.
4. Duplicates.
5. Full text was inaccessible.
6. Other outcomes

Other types of studies not in the scope of the review

### Database and search terms

Databases included in the search were PubMed, Google Scholar, and ScienceDirect. Our search terms included: perioperative, intensive care, HTN, HPT, and BP (S1 File).

### Study selection and data extraction

In Round 1, the research team, which consisted of two researchers (MC and SP), reviewed each title and abstract to exclude articles that did not meet the inclusion criteria. An arbitration process then took place for any articles about which there was a disagreement. The research team had to come to a unanimous agreement. Those articles with acceptable titles and abstracts were acquired and reviewed again by both reviewers. Round 1 concluded after full-text arbitration.

In Round 2, the search included all articles that cited or were cited by the Round 1 articles based on a Web of Science search. The titles and abstracts of the citations underwent the same review process as Round 1. The citations with acceptable titles and abstracts were acquired and reviewed again by both reviewers. Round 2 concluded after full-text arbitration.

Data extraction was performed by two researchers and is summarized considering the following study characteristics: study type, author, year, population, sample size, their definition of acute HTN or HPT, and outcomes in terms of morbidity and mortality (probabilities, odds ratio (OR), hazard ratio (HR), and relative risk (RR)), as well as type of intervention, cardiac or non-cardiac surgery. When necessary, more information is provided.

Additionally, the quality of these studies was graded by using the Scottish Intercollegiate Guidelines (SIGN) [10].

## Results

**Fig 2:**
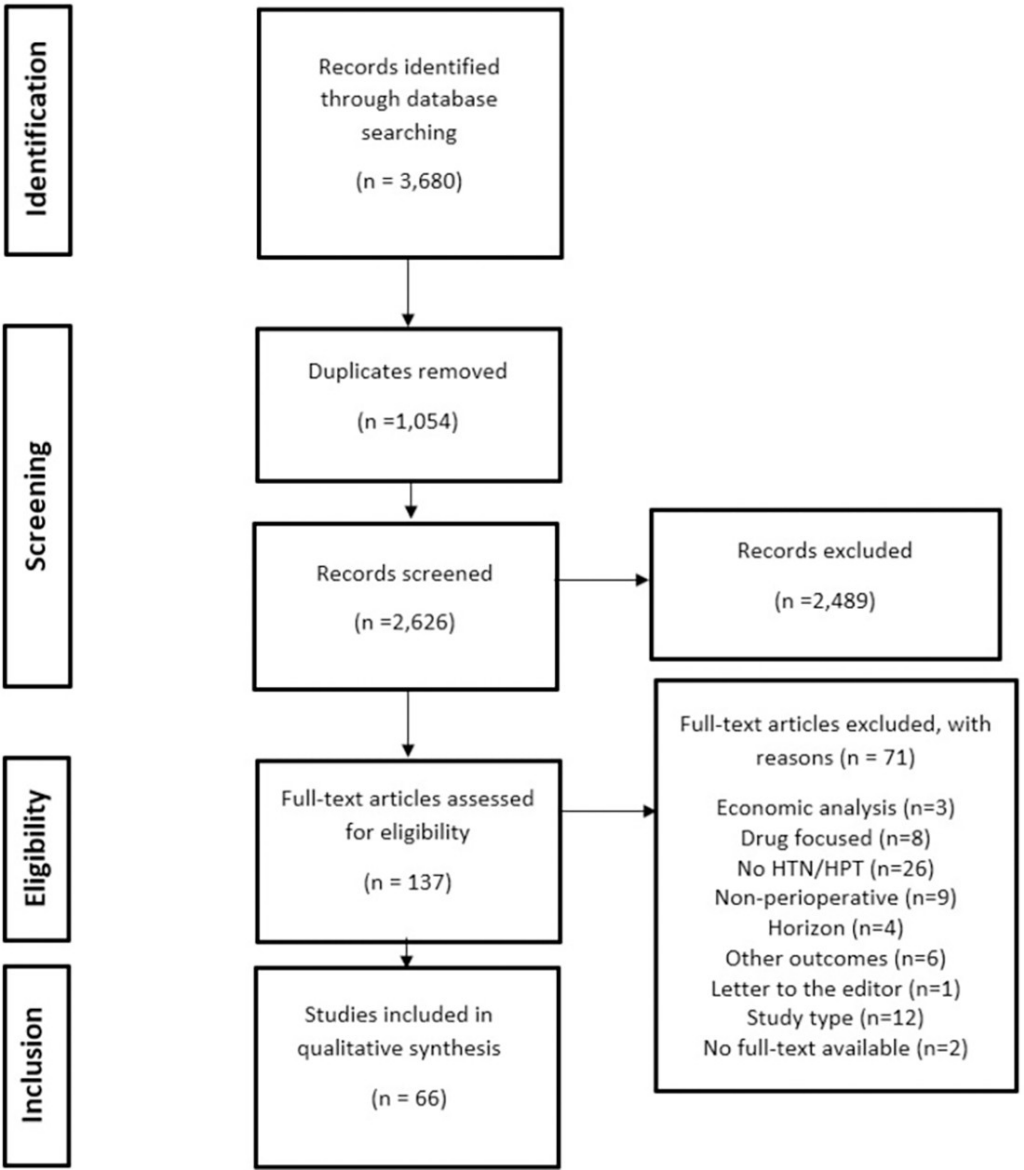
PRISMA diagram.

The literature search led to 3,680 articles being identified, of which, 1,054 were removed for being duplicates. We excluded 2,489 articles based on title and abstract review. We then assessed the full text of 137 articles. After this screening, we identified 66 articles for data extraction. This process is illustrated in the PRISMA diagram (Fig 2). Of these articles, 1 (1.5%) was graded as a moderate-high quality, 39 (59.1%) as moderate, and 26 (39.4%) as low quality (S1 Table).

The study characteristics are shown in Table 1. Sample sizes ranged from 33 to 368,222 patients, with different population types and surgeries. Studies varied greatly in their definition of HTN/HPT. Article results were then grouped by cardiac or non-cardiac surgery, and outcome: mortality, stroke, kidney function, delirium, myocardial injury/AMI, and other outcomes associated with HPT, HTN, or other measures such as control of intraoperative mean arterial pressure (MAP) or variability within the surgery. The parameters were primarily OR, but we also found associations, HR, and RR. For the perioperative setting, the number of articles varies by outcome: 20 mortality, 16 renal outcomes, 6 stroke, 7 delirium, and 34 other outcomes. Since one study may include more than one outcome, the total sum of the reported outcomes may exceed the total number of studies.

**Table 1:** Study characteristics.

Study title	Author	Year	Population	Sample Size	HPT/HTN
**Association between preoperative pulse pressure and perioperative myocardial injury: an international observational cohort study of patients undergoing non-cardiac surgery** [11]	Abbott *et al*.	2017	Non-cardiac surgery	15,057	HTN
**A prospective international multicentre cohort study of intraoperative heart rate and systolic blood pressure and myocardial injury after noncardiac surgery: results of the VISION study** [12]	Abbott *et al*.	2018	Non-cardiac surgery	16,079	HPT/HTN
**Associations of intraoperative radial arterial systolic, diastolic, mean, and pulse pressures with myocardial and acute kidney injury after noncardiac surgery** [13]	Ahuja *et al*.	2020	Non-cardiac surgery	164,514	HPT
**Modifiable, postoperative risk factors for delayed discharge following total knee arthroplasty: the influence of hypotension and opioid use** [14]	Anastasio *et al*.	2020	Total knee arthroplasty	1,033	HPT
**Intraoperative systolic blood pressure variability predicts 30-day mortality in aortocoronary bypass surgery patients** [15]	Aronson *et al*.	2010	Aortocoronary bypass graft surgery	7,504	HPT/HTN
**Does perioperative systolic blood pressure variability predict mortality after cardiac surgery? an exploratory analysis of the ECLIPSE trials** [16]	Aronson *et al*.	2011	Cardiac surgery	1,512	BP variability
**Association between intraoperative low blood pressure and development of surgical site infection after colorectal surgery a retrospective cohort study** [17]	Babazade *et al*.	2016	Colorectal surgery	2,521	HPT
**High postoperative blood pressure after cardiac surgery is associated with acute kidney injury and death** [18]	Balzer *et al*.	2016	Cardiac surgery	5,225	HTN
**Hypotension during hip fracture surgery and postoperative morbidity** [19]	Beecham *et al*.	2020	Hip fracture surgery	52	HPT
**Intraoperative hypotension and perioperative ischemic stroke after general surgery a nested case-control study** [20]	Bijker *et al*.	2012	Non-cardiac and non-neurosurgery	48,241	HPT
**Can routine perioperative haemodynamic parameters predict postoperative morbidity after major surgery?** [21]	Bonnet *et al*.	2020	Non-cardiac surgery	50	HPT
**Association of intraoperative blood pressure instability with adverse outcomes after liver transplantation** [22]	DeMaria *et al*.	2013	Orthotopic liver transplantation	827	HTN
**Intraoperative hypotension is associated with adverse clinical outcomes after noncardiac surgery** [23]	Gregory *et al*.	2020	Non-cardiac surgery	368,222	HPT
**Intraoperative hypotension is associated with myocardial damage in noncardiac surgery: An observational study** [24]	Hallqvist *et al*.	2016	Non-cardiac surgery	300	HPT
**Intraoperative hypotension is associated with acute kidney injury in noncardiac surgery: An observational study**[25]	Hallqvist *et al*.	2018	Non-cardiac surgery	470	HPT
**Impact of intraoperative hypotension and blood pressure fluctuations on early postoperative delirium after non-cardiac surgery** [26]	Hirsch *et al*.	2015	Non-cardiac surgery	594	HPT
**Perioperative optimal blood pressure as determined by ultrasound tagged near infrared spectroscopy and its association with postoperative acute kidney injury in cardiac surgery patients** [27]	Hori *et al*.	2016a	Cardiac surgery	110	HPT
**Blood pressure deviations from optimal mean arterial pressure during cardiac surgery measured with a novel monitor of cerebral blood flow and risk for perioperative delirium: a pilot study** [28]	Hori *et al*.	2016b	Cardiac surgery	110	HPT/HTN
**The association between mild intraoperative hypotension and stroke in general surgery patients** [29]	Hsieh *et al*.	2016	Non-cardiac, non-neurosurgery, and non-carotid surgery	106,337	HPT
**Intraoperative hypotension is a risk factor for postoperative acute kidney injury after femoral neck fracture surgery: a retrospective study** [30]	Jang *et al*.	2019	Femoral neck fracture surgery	248	HPT
**Blood pressure coefficient of variation and its association with cardiac surgical outcomes** [31]	Jinadasa *et al*.	2018	Cardiac surgery	3,687	HTN
**Risk factors for emergence agitation in adults undergoing thoracoscopic lung surgery: a case-control study of 1,950 patients** [32]	Kang *et al*.	2020	Thoracoscopic lung surgery	1,950	HPT/HTN
**Intraoperative hypotension and flap loss in free tissue transfer surgery of the head and neck** [33]	Kass *et al*.	2018	Head and neck surgery	445	HPT
**Intraoperative hypotension is not associated with postoperative cognitive dysfunction in elderly patients undergoing general anesthesia for surgery: results of a randomized controlled pilot trial** [34]	Langer *et al*.	2019	Non-cardiac surgery	101	HPT
**Intraoperative arterial blood pressure lability is associated with improved 30-day survival** [35]	Levin *et al*.	2015	Surgery	52,919	HTN
**Perioperative risk factors associated with acute kidney injury in patients after brain tumor resection** [36]	Li *et al*.	2020a	Brain tumor resection	460	HPT
**High variance of intraoperative blood pressure predicts early cerebral infarction after revascularization surgery in patients with Moyamoya disease** [37]	Li *et al*.	2020b	Revascularization surgery (Moyamoya disease)	1,497	HPT
**Association of intraoperative hypotension with acute kidney injury after liver resection surgery: an observational cohort study** [38]	Liao *et al*.	2020	Liver resection	796	HPT
**Postoperative hypotension after noncardiac surgery and the association with myocardial injury** [39]	Liem *et al*.	2020	Non-cardiac surgery	1,710	HPT
**Perioperative cardiac complications in patients over 80 years of age with coronary artery disease undergoing noncardiac surgery: the incidence and risk factors** [40]	Liu *et al*.	2020	Non-cardiac surgery	547	HPT
**Association between perioperative hypotension and delirium in postoperative critically ill patients: a retrospective cohort analysis** [41]	Maheshwari *et al*.	2019	Non-cardiac surgery	1,083	HPT
**Intraoperative mean arterial pressure variability and 30-day mortality in patients having noncardiac surgery** [42]	Mascha *et al*.	2015	Non-cardiac surgery	104,401	MAP Variability
**Preoperative risk and the association between hypotension and postoperative acute kidney injury** [43]	Mathis *et al*.	2020	Non-cardiac surgery	138,021	HPT
**Prolonged heightened blood pressure following mechanical thrombectomy for acute stroke is associated with worse outcomes** [44]	McCarthy *et al*.	2020	Mechanical thrombectomy	212	HTN
**Association of postoperative blood pressure and bleeding after cardiac surgery** [45]	McIlroy *et al*.	2019	Cardiac surgery	793	HTN
**Relationship between intraoperative hypotension and acute kidney injury after living donor liver transplantation: a retrospective analysis** [46]	Mizota *et al*.	2017	Liver transplantation	231	HPT
**Association between intraoperative hypotension and hypertension and 30-day postoperative mortality in noncardiac surgery** [47]	Monk *et al*.	2015	Non-cardiac surgery	18,756	HPT/HTN
**Postoperative systolic blood pressure as a risk factor for haematoma following thyroid surgery** [48]	Morton and Vandal.	2015	Thyroid surgery	621	HTN
**Defining an intraoperative hypotension threshold in association with de novo renal replacement therapy after cardiac surgery** [49]	Ngu *et al*.	2020	Cardiac surgery	6,523	HPT
**Blood pressure excursions below the cerebral autoregulation threshold during cardiac surgery are associated with acute kidney injury** [50]	Ono *et al*.	2013	Cardiac surgery	348	MAP below limit of autoregulation
**Duration and magnitude of blood pressure below cerebral autoregulation threshold during cardiopulmonary bypass is associated with major morbidity and operative mortality** [51]	Ono *et al*.	2014	Cardiac surgery	450	MAP below limit of autoregulation
**Elevated blood pressure after craniotomy: A prospective observational study** [52]	Perez *et al*.	2020	Craniotomy	282	HTN
**Intraoperative blood pressure changes as a risk factor for anastomotic leakage in colorectal surgery** [53]	Post *et al*.	2012	Colorectal surgery	285	HTN
**Impact of intraoperative hypotension during cardiopulmonary bypass on acute kidney injury after coronary artery bypass grafting** [54]	Rettig *et al*.	2017	Cardiac surgery	1,891	HPT
**The impact of preoperative risk on the association between hypotension and mortality after cardiac surgery: an observational study** [55]	Ristovic *et al*.	2020	Cardiac surgery	6,627	HPT
**Relationship between perioperative hypotension and perioperative cardiovascular events in patients with coronary artery disease undergoing major noncardiac surgery** [56]	Roshanov *et al*.	2019	Non-cardiac surgery	955	HPT
**Intra-operative hypotension is a risk factor for post-operative silent brain ischaemia in patients with pre-operative hypertension undergoing carotid endarterectomy** [57]	Rots *et al*.	2020	Carotid endarterectomy	55	HPT/HTN
**Relationship between Intraoperative Hypotension, defined by either reduction from baseline or absolute thresholds, and acute kidney and myocardial injury after noncardiac surgery** [58]	Salmasi *et al*.	2017	Non-cardiac surgery	57,315	HPT
**Period-dependent associations between hypotension during and for four days after noncardiac surgery and a composite of myocardial infarction and death** [59]	Sessler *et al*.	2018	Non-cardiac surgery	9,765	HPT
**Perioperative hypotension and discharge outcomes in non-critically injured trauma patients, a single centre retrospective cohort study** [60]	Sheffy *et al*.	2017	Trauma surgery	1,744	HPT
**Intraoperative hypotension, new onset atrial fibrillation, and adverse outcome after carotid endarterectomy** [61]	Sposato *et al*.	2011	Carotid endarterectomy	186	HPT
**Association of intraoperative hypotension with acute kidney injury after elective noncardiac surgery** [62]	Sun *et al*.	2015	Non-cardiac surgery	5,127	HPT
**Association of intraoperative hypotension with acute kidney injury after noncardiac surgery in patients younger than 60 years old** [63]	Tang *et al*.	2019	Non-cardiac surgery	4,952	HPT
**Impact of intraoperative hypotension on hospital stay in major abdominal surgery** [64]	Tassoudis *et al*.	2011	Abdominal surgery	100	HPT
**Association between postoperative mean arterial blood pressure and myocardial injury after noncardiac surgery** [65]	van Lier *et al*.	2018	Non-cardiac surgery	2,211	HPT
**Association between intraoperative hypotension and myocardial injury after vascular surgery** [66]	van Waes.	2016	Non-cardiac surgery	890	HPT
**Relationship between intraoperative mean arterial pressure and clinical outcomes after noncardiac surgery: toward an empirical definition of hypotension** [67]	Walsh *et al*.	2013	Non-cardiac surgery	33,330	HPT
**Association between intraoperative blood pressure and postoperative delirium in elderly hip fracture patients** [68]	Wang *et al*.	2015	Hip fracture surgery	103	HTN
**Intraoperative hypotension and delirium after on-pump cardiac surgery** [69]	Wesselink *et al*.	2015	Cardiac surgery	743	HPT
**Concurrence of intraoperative hypotension, low minimum alveolar concentration, and low bispectral index is associated with postoperative death** [70]	Willingham *et al*.	2015	Any surgery	13,198	HPT
**Intraoperative blood pressure variability predicts postoperative mortality in non-cardiac surgery-a prospective observational cohort study** [71]	Wiorek and Krzych.	2019	Non-cardiac surgery	835	BP Variability
**Optimal blood pressure decreases acute kidney injury after gastrointestinal surgery in elderly hypertensive patients: A randomized study Optimal blood pressure reduces acute kidney injury** [72]	Wu *et al*.	2017	Gastrointestinal surgery	678	Control of MAP
**Postoperative hypotension and surgical site infections after colorectal surgery: a retrospective cohort study** [73]	Yilmaz *et al*.	2018	Colorectal surgery	5,896	HPT
**Association of perioperative blood pressure with long-term survival in rectal cancer patients** [74]	Yu *et al*.	2016	Colorectal surgery	358	HTN
**Greater intraprocedural systolic blood pressure and blood pressure variability are associated with contrast-induced neurotoxicity after neurointerventional procedures** [75]	Zevallos *et al*.	2020	Neurointerventions	33	BP Variability
**Perioperative blood pressure control in carotid artery stenosis patients with carotid angioplasty stenting: a retrospective analysis of 173 cases** [76]	Zheng *et al*.	2020	Carotid angioplasty stenting	173	HTN

Abbreviations: BP: blood pressure, HPT: hypotension, HTN: hypertension, MAP: mean arterial pressure.

### Mortality

Twenty articles reporting mortality outcomes in relation to perioperative HPT, HTN, and other measures were retrieved from the search.

#### Hypotension as a risk factor

Ten articles had results for mortality associated with HPT (Table 2). There were two articles focused on cardiac surgery and what these authors demonstrated was that values such as the duration of min excursion of BP <95 mmHg were associated with increased odds (1.03) for 30-day mortality [15], and that MAP below 55 mmHg for more than 10 minutes postsurgery or between 55–64 mmHg intraoperative or postsurgery are also associated with increased odds of death [55]. What was mainly seen in non-cardiac surgeries was that perioperative HPT entailed a higher risk of mortality, with increased odds of 30-day mortality of 1.81 when systolic blood pressure (SBP) was below 100 mmHg, as reported by Abbott *et al*. [12] Other authors also reported increased risk for 30-day mortality with diverse OR such as 1.79 for patients with intraoperative MAP <55 mmHg for more than 20 minutes [67] or 20.826 for intraoperative MAP below 40 mmHg for more than 5 minutes when comparing this patient group with patients with intraoperative BP levels between 60–109 mmHg [47]. The incidence of mortality was reported to be statistically significant for those patients with MAP below 67 mmHg versus above 67.3 mmHg (p<0.001) [65]. Some authors also reported an association between the duration of HPT (measured both as the MAP or SBP) and a higher risk of mortality [47, 67]. In addition, an association between the threshold and the increased odds for 30-day and 90-day mortality has been recently demonstrated: the lower the MAP the higher the odds, as reported by Gregory *et al*. [23].

**Table 2:** Mortality associated with perioperative hypotension.

Author	Year	Sample size	Definition of hypotension	Result*	P-value	Duration
**Aronson *et al***.# [15]	2010	7,504	AUC <95 (mmHg x min)	1.02 (1.002–1.041)	0.03	30-day
			Excursion <95 [AUC for 20% (mmHg x min)]	0.95 (0.916–0.994)	0.02	30-day
			Minutes <95 mmHg per excursion	1.03 (1.008–1.042)	0.003	30-day
			Mean excursion nadir <95 mmHg	1.05 (1.019–1.084)	0.002	30-day
**Ristovic *et al***.# [55]	2020	6,627	MAP 55–64 mmHg 10 min during cardiopulmonary bypass	1.10 (1.00–1.21)	0.049	
			MAP <55 mmHg 10 min post cardiopulmonary bypass	1.30 (1.13–1.49)	0.002	
			MAP 55–64 mmHg 10 min post cardiopulmonary bypass	1.18 (1.07–1.30)	0.001	
**Abbott *et al***. [12]	2018	16,079	SBP <100 mmHg	1.81 (1.39–2.37)	<0.01	30-day
**Gregory *et al***. [23]	2020	368,222	Absolute maximum decrease (for each 5 mmHg under IOH MAP thresholds) 75 mmHg	1.16 (1.14–1.17)		30-day
			Absolute maximum decrease (for each 5 mmHg under IOH MAP thresholds) 65 mmHg	1.21 (1.18–1.23)		30-day
			Absolute maximum decrease (for each 5 mmHg under IOH MAP thresholds) 55 mmHg	1.30 (1.26–1.35)		30-day
			Absolute maximum decrease (for each 5 mmHg under IOH MAP thresholds) 75 mmHg	1.13 (1.12–1.14)		90-day
			Absolute maximum decrease (for each 5 mmHg under IOH MAP thresholds) 65 mmHg	1.17 (1.15–1.20)		90-day
			Absolute maximum decrease (for each 5 mmHg under IOH MAP thresholds) 55 mmHg	1.26 (1.22–1.30)		90-day
**Levin *et al***. [35]	2015	52,919	MAP <50 mmHg 5 min; Derivation cohort (ASA IV or V)	1.19 (1.09–1.29)	<0.001	30-day
			MAP <50 mmHg 5 min; Validation cohort (ASA IV or V)	1.15 (1.04–1.26)	0.01	30-day
**Monk *et al***. [47]	2015	18,756	SBP <70 mmHg for >5 min (Reference 90–159 mmHg)	2.898 (1.719–4.886)	<0.0001	30-day
			MAP 40–49 mmHg for >5 min (Reference 60–109 mmHg)	2.433 (1.285–4.608)	<0.0001	30-day
			MAP <40 mmHg for >5 min (Reference 60–109 mmHg)	20.826 (8.884–48.822)	<0.0001	30-day
			DBP <30 mmHg for >5 min	3.181 (1.826–5.540)	<0.001	30-day
			MBP decrease >50% from baseline for >5 min	2.721 (1.489–4.974)	0.005	30-day
**Sessler *et al***. [59]	2018	9,765	Intra 10-min increase in HPT	1.12 (1.05–1.20)	<0.01	30-day
			Post 10-min increase in HPT	1.03 (1.01–1.06)	<0.01	30-day
**van Lier *et al***. [65]	2018	2,211	MAP 31.0–67.0 mmHg	7% Incidence	<0.001	30-day
			MAP 67.3–76.3 mmHg	2.90% Incidence		
			MAP 76.7–86.0 mmHg	1.90% Incidence		
			MAP 86.7–122.3 mmHg	2.70% Incidence		
**Walsh *et al***. [67]	2013	33,330	MAP <55 mmHg for 1–5 min	1.16 (0.91–1.46)		30-day
			MAP <55 mmHg for 6–10 min	1.16 (0.84–1.60)		30-day
			MAP <55 mmHg for 11–20 min	1.26 (0.89–1.80)		30-day
			MAP <55 mmHg for >20 min	1.79 (1.21–2.65)		30-day
**Willingham *et al***. [70]	2015	13,198	Low MAP 15 min	1.11 (1.08–1.14) HR	<0.001	30-day
			Low MAP 15 min	1.10 (1.08–1.12) HR	<0.001	90-day

Abbreviations: ASA: American Society of Anesthesiologists; AUC: area under the curve, DBP: diastolic blood pressure; CI: confidence interval, IOH: intraoperative hypotension, HPT: hypotension, HR: hazard ratio, MAP: mean arterial pressure, MBP: mean blood pressure, min: minutes, OR: odds ratio, SBP: systolic blood pressure.

*Generally, OR (95% CI), unless otherwise stated.

^#^ These articles refer to cardiac surgery.

#### Hypertension as a risk factor

Ten articles studied mortality in patients with perioperative HTN (Table 3). Four articles investigated the outcomes of cardiac surgery and all but one found statistically significant associations, for example for every 0.10 increase in the coefficient of variation of the SBP an increase in the odds of death of 150% was reported by Jinadasa *et al*. [31], and postoperative HTN (SBP >130 mmHg) was found to be related to higher in-hospital mortality rates [18]. Other authors reported increased odds of 1.03 per minutes above 135 mmHg per BP excursion [15]. One of the articles reported non-statistical significance for the influence of HTN on mortality; the study analyzed the increase of 1 mmHg in SBP [45]. Regarding the non-cardiac surgeries, while some of the studies found an association of HTN and reduced odds of mortality [12, 35], a recent article found increased odds of long-term mortality associated with the maximum SBP in days 2 (OR 1.28) and 3 (OR 1.30) after surgery [44]. One study associated preoperative SBP above 120 mmHg with increased mortality HR in cancer patients [74]. Zheng *et al*. showed that the incidence of in-hospital death was higher in those patients with BP >130 mmHg 24h after the intervention (16.70%, p-value 0.001) [76].

**Table 3:** Mortality associated with perioperative hypertension.

Author	Year	Sample size	Definition of hypertension	Result*	P-value	Duration
**Aronson *et al***.# [15]	2010	7,504	AUC >135 (mmHg x min)	1.02 (0.991–1.055)	0.16	30-day
			Excursions >135 [AUC for 20% (mmHg x min)]	0.99 (0.929–1.058)	0.79	30-day
			Min >135 mmHg per excursion	1.03 (1.001–1.055)	0.04	30-day
			Mean excursion peak >135 mmHg	1.01 (0.989–1.016)	0.14	30-day
**Balzer *et al***.# [18]	2016	5,225	>130 mmHg postoperative		0.001	30-day
**Jinadasa *et al***.# [31]	2018	3,687	SBP coefficient of variation	2.50 (1.60–3.92)	<0.01	30-day
			Pre bypass coefficient of variation	1.75 (1.17–2.62)	0.01	30-day
			Post bypass coefficient of variation	1.40 (0.97–2.04)	0.08	30-day
			MAP coefficient of variation	1.16 (0.98–1.37)	0.09	30-day
			Pre bypass coefficient of variation	1.08 (0.95–1.23)	0.24	30-day
			Bypass coefficient of variation	1.07 (0.87–1.31)	0.53	30-day
			Post bypass coefficient of variation	1.06 (0.85–1.32)	0.63	30-day
**McIlroy *et al***.# [45]	2019	793	SBP 1 mmHg increase	0.98 (0.94–1.02)	0.35	Hospital mortality
			MAP 1 mmHg increase	0.91 (0.84–0.99)	0.03	Hospital mortality
**Abbott *et al***. [12]	2018	16,079	SBP >160 mmHg	0.76 (0.58–0.99)	0.04	30-day
**Levin *et al***. [35]	2015	52,919	Baseline MAP (per 10 mmHg increase); Derivation cohort	0.89 (0.84–0.95)	<0.001	30-day
			Baseline MAP (per 10 mmHg increase); Validation cohort	0.91 (0.84–0.99)	0.03	30-day
			MAP >120 mmHg, per 5 min increase; Derivation cohort	0.91 (0.84–0.99)	0.03	30-day
			MAP >120 mmHg, per 5 min increase; Validation cohort	1.00 (0.93–1.08)	0.98	30-day
**McCarthy *et al***. [44]	2020	212	Maximum SBP day 1 (increases of 10 mmHg)	-		
			Maximum SBP day 2 (increases of 10 mmHg)	1.28 (1.04–1.58)	0.021	Last follow-up (median 89.5 days)
			Maximum SBP day 3 (increases of 10 mmHg)	1.30 (1.06–1.63)	0.015	Last follow-up (median 89.5 days)
			Peak SBP change from day 1 to 2	-		
			Peak SBP change from day 2 to 3	-		
**Monk *et al***. [47]	2015	18,756	SBP >180 mmHg for >5 min (Reference 90–159 mmHg)	0.904 (0.545–1.500)	1	30-day
			MAP >130mmHg for >5 min	0.947 (0.624–1.435)	1	30-day
			DBP >120 mmHg for >5 min	1.357 (0.714–2.580)	1	30-day
**Yu *et al***. [74]	2016	358	Preoperative SBP ≥120 mmHg	1.97 (1.08–3.60) HR	0.028	Disease-free survival
			Preoperative SBP ≥120 mmHg	2.85 (1.00–8.25) HR	0.05	Cancer-free survival
**Zheng *et al***. [76]	2020	173	24h post-CAS BP <120 mmHg	0% Incidence	0.001	In-hospital death
			24h post-CAS BP 120–130 mmHg	0% Incidence		In-hospital death
			24h post-CAS BP >130 mmHg	16.70% Incidence		In-hospital death

Abbreviations: AUC: area under the curve, CAS: carotid angioplasty stenting, CI: confidence interval, DBP: diastolic blood pressure, HR: hazard ratio, MAP: mean arterial pressure, min: minutes, OR: odds ratio, SBP: systolic blood pressure.

*Generally, OR (95% CI), unless otherwise stated.

^#^These articles refer to cardiac surgery.

#### Blood pressure variability

Five articles studied intraoperative BP variability (Table 4) and what was mostly demonstrated was that changes from the baseline of each patient were associated with higher mortality outcomes. Within cardiac surgeries, Aronson *et al*. [16] reported 30-day mortality related to the extent of SBP excursions intraoperatively (outside the range 75–135 mmHg), and pre- and postoperatively (outside the range 85–145 mmHg); another study found that MAP below the limit of autoregulation was also associated with increased odds of mortality in patients undergoing coronary artery bypass grafting and/or valve surgery [51]. The same results were reported for patients undergoing non-cardiac surgery by Mascha *et al*. [42], who found that cumulative time of MAP less than 80, 70, 60, 55, and 50 mmHg were associated with higher odds of 30-day mortality. Small changes, 1% of variability in both DBP and SBP were also related to postoperative mortality [71]. Finally, the control of MAP during surgery between the limits 65–110 mmHg was reported to reduce the risk of mortality [72].

**Table 4:** Mortality associated with perioperative variability in blood pressure (and other measures of blood pressure).

Author	Year	Sample size	Definition of measure	Result*	P-value	Duration	Measure
**Aronson *et al***.# [16]	2011	1,512	SBP, 65–135 mmHg intraoperative 75–145 mmHg pre and post	1.142 (0.989–1.319)	0.0707	30-day	SBP Variability
			SBP, 75–135 mmHg intraoperative 85–145 mmHg pre and post	1.16 (1.039–1.295)	0.0082	30-day	SBP Variability
			SBP, 85–135 mmHg intraoperative 95–145 mmHg pre and post	1.18 (1.076–1.295)	0.0005	30-day	SBP Variability
			SBP, 95–135 mmHg intraoperative 105–145 mmHg pre and post	1.153 (1.074–1.238)	<0.01	30-day	SBP Variability
			SBP, 105–135 mmHg intraoperative 115–145 mmHg pre and post	1.105 (1.053–1.160)	<0.01	30-day	SBP Variability
**Ono *et al***.# [51]	2014	450	AUC _MAP<LLA_ (mmHg x min/h)	1.36 (1.08–1.71)	0.008	Morbidity or mortality	MAP below limit of autoregulation
**Mascha *et al***. [42]	2015	104,401	Average real variability-MAP 25th percentile	1.14 (1.03–1.25)	0.01	30-day	MAP Variability
			Average real variability-MAP median	1.0 (reference)		30-day	MAP Variability
			Average real variability-MAP 75th percentile	0.94 (0.88–0.99)	0.018	30-day	MAP Variability
			10-min sustained minimum MAP (mmHg) minimum MAP <70	0.76 (0.72–0.80)	<0.01	30-day	MAP Variability
			10-min sustained minimum MAP (mmHg) minimum MAP ≥70	1.02 (0.95–1.10)	0.59	30-day	MAP Variability
			Cumulative min of MAP <50 mmHg (10 min)	1.23 (1.15–1.30)	<0.001	30-day	MAP Variability
			Cumulative min of MAP <55 mmHg (10 min)	1.13 (1.09–1.17)	<0.001	30-day	MAP Variability
			Cumulative min of MAP <60 mmHg (10 min)	1.09 (1.07–1.11)	<0.001	30-day	MAP Variability
			Cumulative min of MAP <70 mmHg (10 min)	1.04 (1.03–1.05)	<0.001	30-day	MAP Variability
			Cumulative min of MAP <80 mmHg (10 min)	1.02 (1.01–1.03)	<0.001	30-day	MAP Variability
**Wiorek and Krzych**. [71]	2019	835	SBP variability per 1%	1.10 (1.00–1.21)	0.05	30-day	SBP Variability
			Mean BP variability per 1%	1.10 (0.99–1.23)	0.06	30-day	SBP Variability
			DBP variability per 1%	1.10 (1.01–1.21)	0.03	30-day	SBP Variability
**Wu *et al***. [72]	2017	678	MAP 65 to 79 mmHg	3% Incidence	0.671	28-day mortality incidence	Control of MAP
			MAP 80 to 95 mmHg	2.9% Incidence			Control of MAP
			MAP 96 to 110 mmHg	3.8% Incidence			Control of MAP

Abbreviations: AUC _MAP<LLA_: area under the curve of mean arterial pressure less than the lower limit of autoregulation during the cardiopulmonary bypass, BP: blood pressure, CI: confidence interval, DBP: diastolic blood pressure, MAP: mean arterial pressure, min: minutes, OR: odds ratio, SBP: systolic blood pressure.

*Generally, OR (95% CI), unless otherwise stated.

^#^ These articles refer to cardiac surgery.

### Renal outcomes

Sixteen articles investigated the influence of perioperative HPT and HTN in renal outcomes such as AKI or renal failure.

#### Hypotension as a risk factor

Thirteen articles reported an association between perioperative HPT and increased risk of developing AKI or other adverse renal outcomes (Table 5). There were two works investigating these outcomes in cardiac surgery and it was seen that an area under the curve (AUC) below the optimal BP as well as MAP below 65 mmHg were associated with increased odds of developing AKI and requiring *de novo* renal replacement therapy, respectively. For *de novo* renal replacement therapy higher odds were reported with lower values of MAP [27, 49]. In non-cardiac surgeries an increase of 1.51 odds was associated with a 10% decrease in MAP [46], while other authors demonstrated a cumulative effect of HPT over time, for example Salmasi *et al*. [58], who found increased OR for longer times in the same MAP ranges. The same results were observed by Sun *et al*. [62] for patients with MAP <55 mmHg (11–20 minutes, OR 2.34; >20 minutes, OR 3.53). Tang *et al*. [63] reported OR of 3.25 and 14.11 for patients with MAP <55 mmHg for 11–20 minutes and >20 minutes, respectively. Walsh *et al*. [67] described higher OR with the longest time exposed to MAP <55 mmHg. Mathis *et al*. found statistically significant increased odds for developing AKI when stratifying patients by preoperative risk; odds were increased for high preoperative risk patients with absolute MAP 60–64 mmHg, 55–59 mmHg, 50–54 mmHg, and <50 mmHg, as well as for patients with the highest preoperative risk in the ranges of MAP 55–59 mmHg, 50–54 mmHg, and <50 mmHg; and medium risk patient with MAP below 50 mmHg. These authors reported statistically significant increased odds for low, medium, and high preoperative risk patients when there was a relative decrease in MAP higher than 40% of their baseline MAP [43]. The absolute maximum decrease in intraoperative MAP below 75 mmHg had a statistically significant influence on the risk of needing continuous renal replacement therapy or dialysis, with the risk increasing with each 5 mmHg decrease as reported by Gregory *et al*. [23].

**Table 5:** Acute kidney injury and other renal outcomes associated with perioperative hypotension.

Author	Year	Sample size	Definition of hypotension	Results*	P-value	Injury
**Hori *et al***.# [27]	2016a	110	AUC <optimal BP (MAP with the lowest CFx)	1.03 (1.01–1.05)	0.017	AKI
**Ngu *et al***.# [49]	2020	6,523	MAP (mmHg) per 10 min post cardiopulmonary bypass, <55 mmHg	1.13 (1.05–1.23)	0.002	*De novo* renal replacement therapy
			MAP (mmHg) per 10 min post cardiopulmonary bypass, 55–64 mmHg	1.12 (1.06–1.18)	0.0001	*De novo* renal replacement therapy
			MAP (mmHg) per 10 min post cardiopulmonary bypass, <65 mmHg	1.01 (0.96–1.07)	0.651	*De novo* renal replacement therapy
**Ahuja *et al***. [13]	2020	164,514	AUC under SBP <90 mmHg, Q1 1–21 mmHg x min	0.99 (0.80–1.24)	0.991	AKI
			AUC under SBP <90 mmHg, Q2 22–66 mmHg x min	1.14 (0.92–1.41)	0.15	AKI
			AUC under SBP <90 mmHg, Q3 67–166 mmHg x min	1.09 (0.87–1.35)	0.345	AKI
			AUC under SBP <90 mmHg, Q4 >166 mmHg x min	1.41 (1.14–1.75)	>0.001	AKI
			AUC under MAP <65 mmHg, Q1 1–25 mmHg x min	1.07 (0.84–1.36)	0.474	AKI
			AUC under MAP <65 mmHg, Q2 26–78 mmHg x min	1.19 (0.94–1.50)	0.069	AKI
			AUC under MAP <65 mmHg, Q3 79–198 mmHg x min	1.06 (0.83–1.35)	0.562	AKI
			AUC under MAP <65 mmHg, Q4 >198 mmHg x min	1.43 (1.12–1.82)	<0.001	AKI
			AUC under DBP <50 mmHg, Q1 1–28 mmHg x min	1.07 (0.84–1.36)	0.477	AKI
			AUC under DBP <50 mmHg, Q2 29–99 mmHg x min	1.07 (0.84–1.36)	0.51	AKI
			AUC under DBP <50 mmHg, Q3 100–289 mmHg x min	1.07 (0.83–1.35)	0.562	AKI
			AUC under DBP <50 mmHg, Q4 >289 mmHg x min	1.14 (0.89–1.47)	0.191	AKI
**Gregory *et al***. [23]	2020	368,222	Absolute maximum decrease (for each 5 mmHg under IOH MAP thresholds) 75 mmHg	1.10 (1.07–1.14)		Continuous renal replacement therapy/Dialysis
			Absolute maximum decrease (for each 5 mmHg under IOH MAP thresholds) 65 mmHg	1.12 (1.07–1.17)		Continuous renal replacement therapy/Dialysis
			Absolute maximum decrease (for each 5 mmHg under IOH MAP thresholds) 55 mmHg	1.12 (1.03–1.21)		Continuous renal replacement therapy/Dialysis
**Hallqvist *et al***. [25]	2018	470	The percentage decrease in SBP relative to baseline for more than 5 min (>40 to ≤50%) vs. ≤40%	1.61 (0.99–2.62)	0.054	AKI
			The percentage decrease in SBP relative to baseline for more than 5 min (>50%) vs. ≤40%	2.27 (1.20–4.30)	0.013	AKI
			The percentage decrease in SBP relative to baseline for more than 5 min (>40 to ≤50%) vs. ≤40%	1.48 (0.90–2.44)	0.11	AKI
			The percentage decrease in SBP relative to baseline for more than 5 min (>50%) vs. ≤40%^b^	2.02 (1.05–3.89)	0.034	AKI
**Jang *et al***.[30]	2019	248	SBP <80 mmHg or a mean BP <55–60 mmHg more than 5 min	5.14 (1.54–20.35)	0.012	AKI
**Liao *et al***. [38]	2020	796	Lowest absolute MAP <65 mmHg >10 min during surgery	2.565 (1.271–5.177)	0.009	AKI
**Mathis *et al***. [43]	2020	138,021	Absolute MAP 60–64 mmHg, low preoperative risk	0.96 (0.73–1.27)		AKI
			Absolute MAP 60–64 mmHg, medium preoperative risk	1.12 (0.93–1.36)		AKI
			Absolute MAP 60–64 mmHg, high preoperative risk	1.18 (1.03–1.36)	<0.05	AKI
			Absolute MAP 60–64 mmHg, highest preoperative risk	1.10 (0.99–1.22)		AKI
			Absolute MAP 55–59 mmHg, low preoperative risk	0.90 (0.64–1.27)		AKI
			Absolute MAP 55–59 mmHg, medium preoperative risk	1.18 (0.94–1.47)		AKI
			Absolute MAP 55–59 mmHg, high preoperative risk	1.22 (1.04–1.43)	<0.05	AKI
			Absolute MAP 55–59 mmHg, highest preoperative risk	1.39 (1.25–1.54)	<0.05	AKI
			Absolute MAP 50–54 mmHg, low preoperative risk	1.44 (0.92–2.25)		AKI
			Absolute MAP 50–54 mmHg, medium preoperative risk	1.23 (0.88–1.74)		AKI
			Absolute MAP 50–54 mmHg, high preoperative risk	1.38 (1.08–1.76)	<0.05	AKI
			Absolute MAP 50–54 mmHg, highest preoperative risk	1.57 (1.36–1.83)	<0.05	AKI
			Absolute MAP <50 mmHg, low preoperative risk	1.32 (0.69–2.51)		AKI
			Absolute MAP <50 mmHg, medium preoperative risk	1.77 (1.20–2.61)	<0.05	AKI
			Absolute MAP <50 mmHg, high preoperative risk	1.64 (1.25–2.14)	<0.05	AKI
			Absolute MAP <50 mmHg, highest preoperative risk	2.12 (1.79–2.50)	<0.05	AKI
			MAP 20–30% below baseline, low preoperative risk	1.05 (0.79–1.39)		AKI
			MAP 20–30% below baseline, medium preoperative risk	0.91 (0.74–1.11)		AKI
			MAP 20–30% below baseline, high preoperative risk	0.93 (0.80–1.08)		AKI
			MAP 20–30% below baseline, highest preoperative risk	0.86 (0.78–0.95)		AKI
			MAP 30–40% below baseline, low preoperative risk	1.03 (0.77–1.38)		AKI
			MAP 30–40% below baseline, medium preoperative risk	0.99 (0.81–1.21)		AKI
			MAP 30–40% below baseline, high preoperative risk	1.03 (0.89–1.20)		AKI
			MAP 30–40% below baseline, highest preoperative risk	0.91 (0.82–1.00)		AKI
			MAP >40% below baseline, low preoperative risk	1.48 (1.09–2.00)	<0.05	AKI
			MAP >40% below baseline, medium preoperative risk	1.40 (1.14–1.73)	<0.05	AKI
			MAP >40% below baseline, high preoperative risk	1.24 (1.06–1.45)	<0.05	AKI
			MAP >40% below baseline, highest preoperative risk	1.09 (0.98–1.22)		AKI
**Mizota *et al***. [46]	2017	231	Nadir MAP (per 10 mmHg decrease)	2.11 (1.32–3.47)	0.002	AKI
			Relative decrease in MAP (per 10% decrease)	1.51 (1.11–2.09)	0.01	AKI
			MAP 40–49 mmHg 1–9 min	1.64 (0.49–5.43)	0.419	AKI
			MAP 40–49 mmHg >10 min	2.11 (0.61–7.22)	0.236	AKI
			MAP <40 mmHg 1–9 min	3.80 (1.17–12.30)	0.026	AKI
			MAP <40 mmHg for >10 min	5.06 (1.26–20.40)	0.022	AKI
**Salmasi *et al***. [58]	2017	57,315	Time under MAP <65 mmHg 13–28 min	1.20 (1.02–1.40)	0.0049	AKI
			Time under MAP <65 mmHg >28 min	1.35 (1.14–1.58)	<0.001	AKI
			Time in the lowest MAP categories, 50–55 mmHg 1 min	1.25 (1.02–1.54)	0.0061	AKI
			Time in the lowest MAP categories, 50–55 mmHg 2–4 min	1.29 (1.04–1.61)	0.0029	AKI
			Time in the lowest MAP categories, 50–55 mmHg >4 min	1.43 (1.06–1.92)	0.0031	AKI
			Time in the lowest MAP categories, <50 mmHg 2–4 min	1.23 (1.00–1.50)	0.011	AKI
			Time in the lowest MAP categories, <50 mmHg >4 min	1.43 (1.15–1.78)	<0.0001	AKI
**Sun *et al***. [62]	2015	5,127	MAP <55 mmHg 1–5 min	1.35 (0.98–1.86)		AKI
			MAP <55 mmHg 6–10 min	1.45(0.94–2.22)		AKI
			MAP <55 mmHg 11–20 min	2.34 (1.35–4.05)		AKI
			MAP <55 mmHg >20 min	3.53 (1.51–8.25)		AKI
			MAP <60 mmHg 1–5 min	1.10 (0.70–1.74)		AKI
			MAP<60 mmHg 6–10 min	1.08(0.65–1.78)		AKI
			MAP <60 mmHg 11–20 min	1.84(1.11–3.06)		AKI
			MAP <60 mmHg >20 min	1.70(0.93–3.10)		AKI
			MAP <65 mmHg 1–5 min	1.28 (0.57–2.87)		AKI
			MAP <65 mmHg 6–10 min	1.56(0.69–3.50)		AKI
			MAP <65 mmHg 11–20 min	1.57(0.70–3.53)		AKI
			MAP <65 mmHg >20 min	2.25(0.99–5.07)		AKI
**Tang *et al***. [63]	2019	4,952	MAP <55 mmHg 1–5 min	1.01 (0.51–2)		AKI
			MAP 55–59 mmHg 1–5 min	0.75 (0.35–1.62)		AKI
			MAP 60–64 mmHg 1–5 min	0.53 (0.23–1.24)		AKI
			MAP <55 mmHg 6–10 min	0.96 (0.31–2.96)		AKI
			MAP 55–59 mmHg 6–10 min	1.14 (0.37–3.51)		AKI
			MAP 60–64 mmHg 6–10 min	0.23 (0.06–0.92)	<0.05	AKI
			MAP <55 mmHg 11–20 min	3.25 (1.04–10.14)	<0.05	AKI
			MAP 55–59 mmHg 11–20 min	2.40 (0.85–6.75)		AKI
			MAP 60–64 mmHg 11–20 min	0.40 (0.11–1.47)		AKI
			MAP <55 mmHg >20 min	14.11 (5.02–39.69)	<0.001	AKI
			MAP 55–59 mmHg >20 min	7.46 (3.14–17.72)	<0.001	AKI
			MAP 60–64 mmHg >20 min	2.78 (1.18–6.51)	<0.05	AKI
**Walsh *et al***. [67]	2013	33,330	MAP <55 mmHg 1–5 min	1.18 (1.06–1.31)		AKI
			MAP <55 mmHg 6–10 min	1.19 (1.03–1.39		AKI
			MAP <55 mmHg 11–20 min	1.32 (1.11–1.56)		AKI
			MAP <55 mmHg >20 min	1.51 (1.24–1.84)		AKI

Abbreviations: AUC: area under the curve, AKI: acute kidney injury, CFx: correlation flow index, CI: confidence interval, DBP: diastolic blood pressure, IOH: intraoperative hypotension, MAP: mean arterial pressure, min: minutes, OR: odds ratio, Q: quartile, SBP: systolic blood pressure.

*OR (95%CI),

^#^ These articles refer to cardiac surgery.

#### Hypertension as a risk factor

Jinadasa *et al*. [31] carried out an investigation in 3,687 patients undergoing cardiac surgery about HTN through the coefficient of variation (CV) of both SBP and MAP in cardiac surgical outcomes. In this study, the CV of SBP was found to be a predictor of renal failure [104% increased odds; OR 2.04 (95%CI 1.33–3.14), p-value: 0.001], as well as the CV during the prebypass [OR 1.59 (95%CI 1.07–2.37), p-value: 0.02] and postbypass phases [OR 1.56 (95%CI 1.09–2.22), p-value:0.010]. No other measure was found to be associated with the prediction of renal failure.

#### Control of MAP

A MAP below the lower limit of autoregulation monitored during cardiopulmonary bypass as calculated by a continuous, moving Pearson’s correlation coefficient between MAP and processed near-infrared spectroscopy signals to generate the variable cerebral oximetry index, was reported to increase the relative risk of AKI in cardiac surgical patients [relative risk 1.02 (95%CI 1.01–1.03), p-value<0.0001] in a study carried out by Ono *et al*. that included 348 patients [50]. And the intraoperative control of MAP in the range 80–95 mmHg could reduce postoperative AKI in elderly hypertensive patients (n = 678) after major abdominal surgery according to Wu *et al*. being the incidence (SD) of 31% (13.5%) in patients with a MAP between 65–79 mmHg; 13% (6.3%) in patients with MAP between 80–95 mmHg, and 27% (12.9%) in patients with MAP between 96–110 mmHg, p-value = 0.033 [72].

### Stroke

Six of the retrieved articles investigated the relationship between HPT, HTN, and control of MAP during surgery and stroke.

#### Hypotension as a risk factor

Four articles that studied the relationship between intraoperative HPT and stroke were identified (Table 6) and none of their results found a statistically significant difference in the influence of perioperative HPT on stroke. The approaches included absolute SBP below 100, 90, 80, and 70 mmHg; relative decreases of 10, 20, 30, and 40% from baseline; and MAP below 70, 65, and 60 mmHg [20, 29].

**Table 6:** Stroke associated with intraoperative hypotension.

Author	Year	Sample size	Definition of hypotension	Results*	P-value
**Bijker *et al***. [20]	2012	48,241	Absolute SBP <100 mmHg	1.005 (0.993–1.016)	0.205
			Absolute SBP <90 mmHg	1.006 (0.991–1.022)	0.182
			Absolute SBP <80 mmHg	1.007 (0.981–1.034)	0.368
			Absolute SBP <70 mmHg	1.002 (0.952–1.051)	0.918
			SBP percentage decrease from baseline >10%	1.010 (0.997–1.023)	0.01
			SBP percentage decrease from baseline >20%	1.010 (0.999–1.022)	0.003
			SBP percentage decrease from baseline >30%	1.010 (0.999–1.022)	0.003
			SBP percentage decrease from baseline >40%	1.011 (0.996–1.025)	0.02
			Absolute mean BP <70 mmHg	1.003 (0.993–1.014)^α^	0.296
			Absolute mean BP <60 mmHg	1.003 (0.988–1.014)^α^	0.468
			Absolute mean BP <50 mmHg	1.004 (0.962–1.046)^α^	0.755
			Absolute mean BP <40 mmHg	1.013 (0.939–1.088)^α^	0.563
			Mean BP decrease from baseline >10%	1.004 (0.991–1.016)^α^	0.365
			Mean BP decrease from baseline >20%	1.008 (0.996–1.021)^α^	0.028
			Mean BP decrease from baseline >30%	1.013 (1.000–1.025)^α^	<0.001
			Mean BP decrease from baseline >40%	1.015 (0.999–1.031)^α^	0.003
**Gregory *et al***. [23]	2020	368,222	Absolute maximum decrease (for each 5 mmHg under IOH MAP thresholds) 75 mmHg	1.00 (0.97–1.02)	
			Absolute maximum decrease (for each 5 mmHg under IOH MAP thresholds) 65 mmHg	1.00 (0.96–1.04)	
			Absolute maximum decrease (for each 5 mmHg under IOH MAP thresholds) 55 mmHg	1.02 (0.95–1.09)	
**Hsieh *et al***. [29]	2016	106,337	MAP <70 mmHg	0.49 (0.18–1.38)	
			MAP <70 mmHg	1.07 (0.76–1.53)^β^	
			MAP <65 mmHg	0.68 (0.27–1.71)	
			MAP <65 mmHg	1.16 (0.76–1.77)^β^	
			MAP <60 mmHg	0.72 (0.24–2.17)	
			MAP <60 mmHg	1.26 (0.73–2.19)^β^	
**Li *et al***. [37]	2020b	1,497	Average real variance-SBP (mmHg/min)	3.18 (1.32–10.30)^α^	0.002
			Average real variance-DBP (mmHg/min)	4.04 (1.04–16.82)^α^	0.006
			Average real variance-MAP (mmHg/min)	4.02 (1.22–17.46)^α^	0.004
			Maximum drop-DBP (mmHg)	1.08 (1.02–1.15)^α^	0.001
			Maximum drop-MAP (mmHg)	1.06 (1.00–1.12)^α^	0.006
			Minimal SBP (mmHg)	1.03 (0.99–1.08)^α^	0.048
			Time weighted average-SBP (mmHg)	1.06 (1.02–1.11)^α^	<0.001
			Time weighted average-MAP (mmHg)	1.06 (1.00–1.12)^α^	0.008
			Relative SBP-intraoperative HPT duration, <60% baseline	1.02 (1.00–1.04)^α^	0.037
			Average real variance-SBP (mmHg/min) in massive infarction	5.83 (0.77–52.91)^α^	0.025
			Average real variance-DBP (mmHg/min) in massive infarction	12.90 (0.69–259.94)^α^	0.021
			Average real variance-MAP (mmHg/min) in massive infarction	20.22 (1.08–578.16)^α^	0.011
			Maximum drop-DBP (mmHg)	1.10 (1.01–1.22)^α^	0.006
			Maximum drop-MAP (mmHg)	1.11 (1.01–1.22)^α^	0.005

Abbreviations: BP: blood pressure, CI: confidence interval, DBP: diastolic blood pressure, HPT: hypotension, IOH: intraoperative hypotension, MAP: mean arterial pressure, min: minutes, OR: odds ratio, SBP: systolic blood pressure.

*Generally, OR (95% CI), unless otherwise stated;

^α^, OR adjusted (99.9% CI);

^β^, OR (Ratio of Geometric Mean).

#### Hypertension as a risk factor

Zheng *et al*. [76] investigated the influence of baseline and 24h after intervention SBP on the incidence of stroke in 173 patients undergoing carotid angioplasty stenting (CAS). Baseline SBP was not found to be statistically associated with stroke incidence being the incidence 0% in those patients with baseline SBP <120 mmHg, 7.90% for SBP 120–130 mmHg, and 4.10% for SBP >130 mmHg; p-value: 0.41. Post-intervention SBP increases incidence, increasing proportionally with higher levels of SBP, but no statistical significance was found; 3.50% incidence in patients with 24h post-CAS SBP <120 mmHg, 7.50% for 24h post-CAS SBP 120–130 mmHg, and 11.10% in 24h post-CAS SBP >130 mmHg; p-value = 0.174.

#### Control of MAP

In the study performed by Wu *et al*. in 678 patients, there was no statistically significant difference in stroke after gastrointestinal surgery in elderly patients whose ranges of MAP were 65–79 mmHg (0.43% incidence), 80–95 mmHg (0.48% incidence), and 96–110 mmHg (0.47%); p-value = 0.341 [72].

### Delirium

There were seven articles reporting results for the association of perioperative HPT, HTN and postsurgical delirium.

#### Hypotension as a risk factor

Studies investigating the effect of perioperative HPT on delirium (n = 5) (Table 7) found that what might be related to the development of postsurgical delirium is BP fluctuation or variance, the so-called lability, and no absolute or relative values of HPT themselves both in cardiac and non-cardiac surgical patients [26]. In those articles studying cardiac surgeries, influence of HPT over delirium was demonstrated [28, 69]. Additionally, other authors studying non-cardiac interventions, reported increased HR for delirium in those patients with intraoperative and postoperative HPT (during intensive care unit (ICU) stay) [41] and increased OR of delirium for decreases of intraoperative MAP in ranges below 75 mmHg [23]. The study by Hirsch *et al*. [26] reported increased OR for the prediction of the number of days of delirium, both with MAP (OR 1.038, 95% CI 1.010–1.067, p-value = 0.008) and SBP (OR 1.018 95% CI 1.005–1.030, p-value = 0.004) variance as measured by mmHg^2^ per 10 units decrease. Another study found no statistically significant results for the association of HPT with postoperative delirium in elderly patients undergoing non-cardiac surgery [34].

**Table 7:** Delirium associated with perioperative hypotension.

Author	Year	Sample size	Definition of hypotension	Results*	P-value
**Hori *et al***.# [28]	2016b	110	AUC <optimal MAP (mmHg x h) overall	Incidence	0.7
			AUC <optimal MAP (mmHg x h) postoperative day 1	Incidence	0.5
			AUC <optimal MAP (mmHg x h) postoperative day 2	Incidence	0.61
			AUC <optimal MAP (mmHg x h) postoperative day 3	Incidence	0.82
**Wesselink *et al***.# [69]	2015	743	MAP <60 mmHg	1.04 (0.99–1.10)^α^	0.04
			MAP <50 mmHg	1.22 (1.02–1.66)^α^	0.04
			MAP decrease >30% relative to baseline	1.00 (0.99–1.01)^α^	0.45
			MAP decrease >40% relative to baseline	1.00 (0.99–1.02) ^α^	0.55
**Gregory *et al***. [23]	2020	368,222	Absolute maximum decrease (for each 5 mmHg under IOH MAP thresholds) 75 mmHg	1.04 (1.01–1.07)	
			Absolute maximum decrease (for each 5 mmHg under IOH MAP thresholds) 65 mmHg	1.05 (1.01–1.10)	
			Absolute maximum decrease (for each 5 mmHg under IOH MAP thresholds) 55 mmHg	1.07 (1.00–1.15)	
**Langer *et al***. [34]	2019	101	MAP ≥90% time below of preoperative values		0.19
**Maheshwari *et al***. [41]	2019	1,083	Intraoperative period: TWA of MAP <65 mmHg	1.11 (1.03–1.20) HR	0.009
			ICU stay: Lowest MAP on each day (10 mmHg decrease in lowest MAP)	1.12 (1.04–1.20) HR	0.003
			MAP <50 mmHg	1.14 (0.98–1.53)^α^	0.09

Abbreviations: AUC: area under the curve, CI: confidence interval, HR: hazard ratio, ICU: intensive care unit, IOH: intraoperative hypotension; MAP: mean arterial pressure, OR: odds ratio, SBP: systolic blood pressure, TWA: time-weighted average.

*Generally, OR (95% CI), unless otherwise stated in the Outcome type column.

^#^ These articles refer to cardiac surgery.

^α^, OR adjusted (99% CI).

#### Hypertension as a risk factor

Two different studies demonstrated the implication of intraoperative HTN for postsurgical delirium. (Table 8) Hori *et al*. [28] found a statistically significant association of the AUC above the optimal MAP (as measured in mmHg x h) during cardiac surgery and the development of delirium on postoperative day 2, and the other reported increased odds (2.34) for delirium when suffering an increase of 10 mmHg in mean surgery mean arterial pressure (msMAP) when msMAP was equal or above 80 mmHg during trauma surgery [68].

**Table 8:** Delirium associated with perioperative hypertension.

Author	Year	Sample size	Definition of HT	Results*	P-value	Time
**Hori *et al***.# [28]	2016b	110	AUC >optimal MAP (mmHg x h) overall	Incidence	0.4	Overall
			AUC >optimal MAP (mmHg x h) postoperative day 1	Incidence	0.12	POD 1
			AUC >optimal MAP (mmHg x h) postoperative day 2	Incidence	0.031	POD 2
			AUC >optimal MAP (mmHg x h) postoperative day 3	Incidence	0.59	POD 3
**Wang *et al***. [68]	2015	103	Per 10 mmHg increase of msMAP if msMAP was <80 mmHg	0.21 (0.05–0.86)	0.03	
			Per 10 mmHg increase of msMAP if msMAP was ≥80 mmHg	2.34 (1.11–4.94)	0.03	
			MAP value at baseline	1.44 (0.92–2.25)	0.11	

Abbreviations: AUC: area under the curve, CI: confidence interval, HT: hypertension, ICU: intensive care unit, msMAP: average MAP during surgery, MAP: mean arterial pressure, OR: odds ratio, POD: postoperative day, SBP: systolic blood pressure.

*Generally, OR (95% CI), unless otherwise stated,

^#^ This article refers to cardiac surgery.

### Cardiovascular and cerebrovascular outcomes

There were 18 articles reporting results for the association of HPT and HTN with cardiovascular and cerebrovascular adverse outcomes such as AMI, myocardial injury after non-cardiac surgery (MINS), and others.

#### Hypotension as a risk factor

HPT association with cardiovascular and cerebrovascular outcomes was investigated in 15 articles (Table 9). One of them reported results from patients undergoing cardiac surgery. The work by Sposato *et al*. [61] described the new onset of atrial fibrillation associated with intraoperative SBP below 80 mmHg for 15 minutes or more with increased odds of 9.6 in cardiac surgery. The rest of the articles referred to non-cardiac surgery; some of them studied HPT in relation to various cardiac outcomes and found a statistically significant association between SBP <100 mmHg and myocardial injury, increasing with an OR of 1.21 [12], this relation of HPT was also seen when the decrease in SBP is more than 50% relative to baseline values for more than 5 minutes, with OR of 4.4 for myocardial damage [24]. Other studies such as that of Roshanov *et al*. [56] reported that not just the intraoperative HPT is relevant for the development of cardiovascular events (SBP <90 mmHg for more than 10 minutes, OR 2.43) but also postoperative HPT (SBP <90 mmHg for more than 10 minutes, OR 2.17); there was also evidence showing that in addition to the postoperative MAP, the time under different thresholds also has an influence on the development of AMI [39]. Additionally it has been published that time under MAP also has an influence on myocardial outcomes, as demonstrated by Salmasi *et al*. [58], odds for MINS (AMI after non-cardiac surgery) MAP <65 mmHg during 13–28 minutes were 1.34, while odds for MAP <65 mmHg during more than 28 minutes were 1.60; influence was also seen for MAP <50 mmHg, in this category just one minutes below 50 mmHg was found to be statistically significant associated with the development of MINS. As well as Salmasi *et al*., van Lier *et al*. [65] saw that there was a statistically significant relationship between the levels of HPT and the incidence of AMI, with the relationship being inversely proportional. Walsh *et al*. [67] also found that a longer time below <55 mmHg had an impact on the greater odds for both myocardial injury and cardiac complication. Gregory *et al*. reported statistically significant increased odds for developing MACCE (composite measure of all-cause mortality, AMI, or acute ischemic stroke) by any measure of MAP under any threshold considered, they also found increased odds for AMI [23].

**Table 9:** Cardiovascular and cerebrovascular outcomes associated with perioperative hypotension.

Author	Year	Sample size	Definition of hypotension	Result*	P-value	Event
**Sposato *et al***.# [61]	2011	186	Intraoperative SBP <80 mmHg ≥15 min	9.6 (1.9–47.4)	0.006	New onset atrial fibrillation
**Abbott *et al***. [12]	2018	16,079	SBP <100 mmHg	1.21 (1.05–1.39)	0.01	Myocardial injury
			SBP <100 mmHg	1.21 (0.98–1.49)	0.07	AMI
**Ahuja *et al***. [13]	2020	164,514	AUC under SBP <90 mmHg, Q1 1–21 mmHg x min	0.85 (0.65–1.10)^γ^	0.107	MINS
			AUC under SBP <90 mmHg, Q2 22–66 mmHg x min	1.00 (0.78–1.29) ^γ^	0.974	MINS
			AUC under SBP <90 mmHg, Q3 67–166 mmHg x min	1.16 (0.91–1.49) ^γ^	0.122	MINS
			AUC under SBP <90 mmHg, Q4 >166 mmHg x min	1.65 (1.30–2.09) ^γ^	>0.001	MINS
			AUC under MAP <65 mmHg, Q1 1–25 mmHg x min	1.03 (0.78–1.38) ^γ^	0.766	MINS
			AUC under MAP <65 mmHg, Q2 26–78 mmHg x min	1.09 (0.82–1.46) ^γ^	0.438	MINS
			AUC under MAP <65 mmHg, Q3 79–198 mmHg x min	1.37 (1.04–1.81) ^γ^	0.004	MINS
			AUC under MAP <65 mmHg, Q4 >198 mmHg x min	1.51 (1.14–2.00) ^γ^	<0.001	MINS
			AUC under DBP <50 mmHg, Q1 1–28 mmHg x min	1.01 (0.75–1.35) ^γ^	0.956	MINS
			AUC under DBP <50 mmHg, Q2 29–99 mmHg x min	1.15 (0.86–1.54) ^γ^	0.223	MINS
			AUC under DBP <50 mmHg, Q3 100–289 mmHg x min	1.20 (0.90–1.60) ^γ^	0.117	MINS
			AUC under DBP <50 mmHg, Q4> 289 mmHg x min	1.23 (0.91–1.65)	0.081	MINS
**Gregory *et al***. [23]	2020	368,222	Absolute maximum decrease (for each 5 mmHg under IOH MAP thresholds) 75 mmHg	1.12 (1.11–1.14)	<0.001	MACCE
			Absolute maximum decrease (for each 5 mmHg under IOH MAP thresholds) 65 mmHg	1.17 (1.15–1.19)	<0.001	MACCE
			Absolute maximum decrease (for each 5 mmHg under IOH MAP thresholds) 55 mmHg	1.26 (1.22–1.29)	<0.001	MACCE
			TWA-MAP (75 mmHg)	1.03 (1.03–1.04)	<0.001	MACCE
			TWA-MAP (65 mmHg)	1.07 (1.06–1.08)	<0.001	MACCE
			TWA-MAP (55 mmHg)	1.14 (1.12–1.16)	<0.001	MACCE
			Absolute cumulative time (75 mmHg)	1.02 (1.02–1.03)	<0.001	MACCE
			Absolute cumulative time (65 mmHg)	1.03 (1.03–1.04)	<0.001	MACCE
			Absolute cumulative time (55 mmHg)	1.05 (1.04–1.06)	<0.001	MACCE
			Absolute maximum decrease (for each 5 mmHg under IOH MAP thresholds) 75 mmHg	1.07 (1.04–1.10)		AMI
			Absolute maximum decrease (for each 5 mmHg under IOH MAP thresholds) 65 mmHg	1.10 (1.05–1.14)		AMI
			Absolute maximum decrease (for each 5 mmHg under IOH MAP thresholds) 55 mmHg	1.12 (1.05–1.21)		AMI
**Hallqvist *et al***. [24]	2016	300	Decrease in SBP more than 50% relative to baseline for more than 5 min	4.4 (1.8–11.1)		Myocardial damage
**Kass *et al***. [33]	2018	445	Baseline MAP, per 10 mmHg increase	0.96 (0.75–1.257)	0.782	Flap loss
			MAP <60 mmHg for >20 episodes, per episode increase	1.22 (1.11–1.35)	<0.01	Flap loss
**Liem *et al***. [39]	2020	1,710	Postoperative MAP <60 mmHg 0-1h	1.54 (1.05–2.24)	0.027	Myocardial injury
			Postoperative MAP <60 mmHg 1-2h	2.62 (1.39–4.80)	0.002	Myocardial injury
			Postoperative MAP <60 mmHg 2-4h	3.26 (1.57–6.48)	0.001	Myocardial injury
			Postoperative MAP <65 mmHg >4h	2.98 (1.78–4.98)	<0.001	Myocardial injury
			Postoperative MAP <70 mmHg >4h	2.18 (1.37–3.51)	0.001	Myocardial injury
			Postoperative MAP <75 mmHg >4h	2.03 (1.19–3.60)	0.012	Myocardial injury
**Liu *et al***. [40]	2020	547	Intraoperative HPT (SBP <90 mmHg or 70% baseline or DBP <60 mmHg lasting at least 10 min)	25.50% Incidence	0.003	Perioperative cardiac complications
**Roshanov *et al***. [56]	2019	955	Any SBP <90 mmHg more than 10 min	3.17 (1.99–5.06) HR	<0.01	Cardiovascular events
			Intraoperative SBP <90 mmHg more than 10 min	2.43 (1.50–3.95) HR	<0.01	Cardiovascular events
			Postoperative SBP <90 mmHg more than 10 min	2.17 (1.35–3.49) HR	0.001	Cardiovascular events
**Rots *et al***. [57]	2020	55	SBP intraoperative—SBP preoperative (mean differences)		0.024	Silent brain ischaemia
**Salmasi *et al***. [58]	2017	57,315	Time under MAP <65 mmHg 13–28 min	1.34 (1.06–1.60)	0.0015	MINS
			Time under MAP <65 mmHg >28 min	1.60 (1.28–2.01)	<0.001	MINS
			Time under MAP <50 mmHg 1 min	1.49 (1.13–1.96)	<0.001	MINS
			Time under MAP <50 mmHg 2–4 min	1.63 (1.26–2.10)	<0.001	MINS
			Time MAP <50 mmHg >4 min	1.97 (1.50–2.59)	<0.001	MINS
**Sessler *et al***. [59]	2018	9,765	Intraoperative 10-min increase in HPT	1.03 (0.97–1.10)	0.162	AMI
			Remaining day of surgery 10min increase in HPT	1.03 (1.00–1.05)	0.002	AMI
**van Lier *et al***. [65]	2018	2,211	MAP 31.0–67.0 mmHg	4.80% Incidence	p = 0.005; OR 0.991 (0.983–0.999)	AMI
			MAP 67.3–76.3 mmHg	2.90% Incidence		AMI
			MAP 76.7–86.0 mmHg	1.40% Incidence		AMI
			MAP 86.7–122.3 mmHg	Incidence		AMI
**van Waes *et al***. [66]	2016	890	MAP <50 mmHg 2–5 min	1.3 (0.8–2.2)^δ^	0.21	Myocardial injury
			MAP <50 mmHg 6–10 min	2.0 (1.1–3.6)^δ^	0.003	Myocardial injury
			MAP <50 mmHg 11–20 min	1.0 (0.4–2.2)^δ^	0.89	Myocardial injury
			MAP <50 mmHg 21–30 min	2.0 (0.8–5.1)^δ^	0.08	Myocardial injury
			MAP <50 mmHg >30 min	1.5 (0.4–6.7)^δ^	0.47	Myocardial injury
			MAP <60 mmHg 2–5 min	1.1 (0.7–1.7)^δ^	0.52	Myocardial injury
			MAP <60 mmHg 6–10 min	0.9 (0.5–1.6)^δ^	0.58	Myocardial injury
			MAP <60 mmHg 11–20 min	1.5 (1.0–2.3)^δ^	0.02	Myocardial injury
			MAP <60 mmHg 21–30 min	1.5 (1.0–2.5)^δ^	0.02	Myocardial injury
			MAP <60 mmHg >30 min	1.7 (1.1–2.6)^δ^	0.004	Myocardial injury
**Walsh *et al***. [67]	2013	33,330	MAP <55 mmHg 1–5 min	1.30 (1.06–1.58)		Myocardial injury
			MAP <55 mmHg 6–10 min	1.47 (1.13–1.93)		Myocardial injury
			MAP <55 mmHg 11–20 min	1.79 (1.33–2.39)		Myocardial injury
			MAP <55 mmHg > 20 min	1.82 (1.31–2.55)		Myocardial injury
			MAP <55 mmHg 1–5 min	1.35 (1.15–1.58)		Cardiac complication
			MAP <55 mmHg 6–10 min	1.46 (1.17–1.83)		Cardiac complication
			MAP <55 mmHg 11–20 min	1.50 (1.16–1.94)		Cardiac complication
			MAP <55 mmHg >20 min	1.95 (1.46–2.60)		Cardiac complication

Abbreviations: AMI: acute myocardial infarction, DBP: diastolic blood pressure, HPT: hypotension, HR: hazard ratio, IOH: intraoperative hypotension, MACCE: composite measure of all-cause mortality, AMI, or acute ischemic stroke, MAP: mean arterial pressure, min: minutes, MINS: myocardial infarction after non-cardiac surgery, OR: odds ratio, SBP: systolic blood pressure, TWA: time-weighted average.

*Generally, OR (95% CI), unless otherwise stated in the Outcome type column.

^#^ This article refers to cardiac surgery.

^γ^, OR adjusted (98.75% CI);

^δ^, RR (98.8% CI).

#### Hypertension as a risk factor

Studies investigating the relationship between HTN and cardiac or cerebrovascular outcomes in non-cardiac surgery were also retrieved from our search (n = 5) (Table 10). Abbott *et al*. [12] described a statistically significant association between SBP above 160 mmHg and myocardial injury and AMI, which had been found to not be associated in a previous study [11]. Outcomes such as silent brain ischemia were found to be statistically significant related to a 20% increase of baseline SBP during carotid endarterectomy [57]. Further evidence regarding the influence of postoperative HTN has been described by Zheng *et al*. [76], who reported increased incidence of intracranial hemorrhage and unfavorable discharge rising with higher ranges of postoperative SBP; these authors report increased OR of intracranial hemorrhage when SBP postsurgery is above 130 mmHg [OR 37.67 (6.79–209.01), p<0.0001].

**Table 10:** Cardiac and cerebrovascular outcomes associated with perioperative hypertension.

Author	Year	Sample size	Definition of hypertension	Result*	P-value	Event
**Abbott *et al***. [11]	2017	15,057	SBP <120 mmHg	1.07 (0.91–1.24)	0.43	Myocardial injury
			SBP 120–131 mmHg	1.19 (0.97–1.44)	0.09	Myocardial injury
			SBP 132–143 mmHg	0.95 (0.83–1.10)	0.52	Myocardial injury
			SBP 144–159 mmHg	0.93 (0.80–1.07)	0.29	Myocardial injury
			SBP ≥160 mmHg	0.90 (0.75–1.07)	0.24	Myocardial injury
**Abbott *et al***. [12]	2018	16,079	SBP >160 mmHg	1.16 (1.01–1.34)	0.04	Myocardial injury
			SBP >160 mmHg	1.34 (1.09–1.64)	0.01	AMI
**Pérez *et al***. [52]	2020	282	Post craniotomy severe HTN (SBP ≥170 mmHg)	0% Incidence		Intracerebral haemorrhage
			Post craniotomy severe HTN (SBP ≥170 mmHg)	2.70% Incidence		Ischemic stroke
**Rots *et al***. [57]	2020	55	SBP intraoperative increase ≥20% of SBP preoperative (duration of relative HTN)		0.048	Silent brain ischaemia
**Zheng *et al***. [76]	2020	173	Baseline SBP <120 mmHg	4.20% Incidence	0.803	Intracranial haemorrhage
			Baseline SBP 120–130 mmHg	7.90% Incidence		Intracranial haemorrhage
			Baseline SBP >130 mmHg	9.60% Incidence		Intracranial haemorrhage
			24h post-CAS SBP <120 mmHg	2.60% Incidence	<0.0001	Intracranial haemorrhage
			24h post-CAS SBP 120–130 mmHg	5.00% Incidence		Intracranial haemorrhage
			24h post-CAS SBP >130 mmHg	50% Incidence		Intracranial haemorrhage
			24h post-CAS SBP >130 mmHg	37.67 (6.79–209.01)	<0.0001	Intracranial haemorrhage

Abbreviations: AMI: acute myocardial infarction, CAS: carotid angioplasty stenting, HTN: hypertension, SBP: systolic blood pressure.

*OR (95% CI).

### Other outcomes

In total, 16 articles were retrieved reporting other types of outcomes associated with perioperative HPT or HTN.

#### Hypotension as a risk factor

Nine of them investigated perioperative HPT and other relevant outcomes such as myocardial injury or surgical site infection (Table 11). Other studies such as that of Roshanov *et al*. [56] reported hypotensive events in postoperative days 0 and 1 was also found to be associated with increased length of hospital stay (OR 1.305, p = 0.0239) [14, 21]; and sepsis/systemic inflammatory response syndrome [23]. Other outcomes that were investigated were: surgical site infection, that was found to be not statistically significant in relation to MAP <55 mmHg and SBP <80 mmHg, but odds of 1.08 were found in association with minimum postoperative MAP, per 5 mmHg decrease by Yilmaz *et al*. [73]; or flap loss that was associated with MAP <60 mmHg for more than 20 episodes and per each episode the odds were 1.22 [33]. Increased risk of poor discharge outcomes (defined as death, discharge to a nursing facility, or discharge to hospice care) were reported by Sheffy *et al*. [60] for those patients with perioperative SBP <90 mmHg or <110 mmHg undergoing trauma surgery. Tassoudis *et al*. [64] described increased odds for hospital stay, complications, and duration of complications related to intraoperative MAP below 60 mmHg or MAP below 70 mmHg and MAP decrease above 30% from baseline values. Emergence agitation was associated with SBP <90 mmHg intraoperatively (OR 1.636, p = 0.025) in patients undergoing thoracoscopic lung surgery [32].

**Table 11:** Diverse outcomes associated with perioperative hypotension.

Author	Year	Sample size	Definition of hypotension	Result*	P-value	Event
**Anastasio *et al***. [14]	2020	1,033	Hypotensive events (SBP <90 mmHg or DBP <60 mmHg for any single reading) on POD 0/1	1.305 (1.036–1.645)	0.0239	LoS >3 days
**Babazade *et al***. [17]	2016	2,521	MAP <55 mmHg	0.97 (0.81–1.17)	0.71	Surgical site infection
			SBP <80 mmHg	0.96 (0.84–1.11)	0.54	Surgical site infection
			Cumulative min of MAP <55 mmHg	0.97 (0.91–1.04) HR	0.36	Time to discharge alive
			Cumulative min of SBP <80 mmHg	0.97 (0.93–1.01) HR	0.13	Time to discharge alive
**Beecham *et al***. [19]	2020	52	1-min increase cumulative time of SAP <80% pre-induction	1.020 (1.008–1.035)	0.003	Clavien-Dindo (progression to a higher level)
**Bonnet *et al***. [21]	2020	50	SBP <100 mmHg (duration)		0.38	Comprehensive Complications Index
			MAP <75 mmHg (duration)		0.03	Comprehensive Complications Index
			MAP <55 mmHg (duration)		0.11	Comprehensive Complications Index
			SBP <100 mmHg (duration)		0.35	LoS
			MAP <75 mmHg (duration)		0.03	LoS
			MAP <55 mmHg (duration)		0.53	LoS
**Gregory *et al***. [23]	2020	368,222	Absolute maximum decrease (for each 5 mmHg under IOH MAP thresholds) 75 mmHg	1.15 (1.12–1.18)		Sepsis/Systemic inflammatory response syndrome
			Absolute maximum decrease (for each 5 mmHg under IOH MAP thresholds) 65 mmHg	1.21 (1.16–1.26)		Sepsis/Systemic inflammatory response syndrome
			Absolute maximum decrease (for each 5 mmHg under IOH MAP thresholds) 55 mmHg	1.28 (1.20–1.37)		Sepsis/Systemic inflammatory response syndrome
**Kang *et al***. [32]	2020	1,950	Intraoperative hypotension (SBP < 90 mmHg assessed more than 5 min apart)	1.636 (1.064–2.514)	0.025	Emergence agitation
**Sheffy *et al***. [60]	2017	1,744	Perioperative SBP <90 mmHg	1.55 (1.04–2.31) RR	<0.05	Poor discharge outcome
			Perioperative SBP <110 mmHg	1.87 (1.17–2.98) RR	<0.01	Poor discharge outcome
**Tassoudis *et al***. [64]	2011	100	Intraoperative MAP <60 mmHg or MAP <70 mmHg and MAP decrease >30% compared to baseline value	4.56 (1.85–10.96)	0.001	LoS
			Intraoperative MAP <60 mmHg or MAP <70 mmHg and MAP decrease >30% compared to baseline value	5.10 (1.95–13.35)	0.001	Complications
			Intraoperative MAP <60 mmHg or MAP <70 mmHg and MAP decrease >30% compared to baseline value	4.54 (1.88–10.92)	0.001	Duration of complications
**Yilmaz *et al***. [73]	2018	5,896	Postoperative TWA-MAP Quintile (mmHg) [OR for a 5-mmHg decrease in TWA-MAP or minimum MAP]	1.03 (0.99–1.08)	0.16	Surgical site infection
			Minimum postoperative MAP (per 5 mmHg decrease)	1.08 (1.03–1.12)	0.001	Surgical site infection

Abbreviations: DBP: diastolic blood pressure, HR: hazard ratio, IOH: intraoperative hypotension, LoS: length of hospital stay, MAP: mean arterial pressure, min: minutes, POD: postoperative day, RR: risk ratio, SAP: systolic arterial pressure, SBP: systolic blood pressure, TWA: time-weighted average.

*Generally, OR (95%CI), unless otherwise stated in the Outcome type column.

#### Hypertension as a risk factor

There were eight studies investigating the relationship between HTN and various outcomes that were retrieved from our search (Table 12). Other outcomes have been associated with HTN as well: hematoma [48], emergence agitation [32], and anastomotic leakage [53]. Postoperative HTN was reported to have a negative impact on functional independence of patients undergoing mechanical thrombectomy, especially for those patients with maximum SBP in postoperative day (POD) 3 [44]. There were two articles on cardiac surgical patients and one of the studies found statistically significant increased odds for the length of hospital stay due to HTN [18].

**Table 12:** Diverse outcomes associated with perioperative hypertension.

Author	Year	Sample size	Definition of hypertension	Result*	P-value	Event
**Balzer *et al***.# [18]	2016	5,225	SBP >130 mmHg		0.119	LoS in ICU
			SBP >130 mmHg		0.024	LoS in hospital
			SBP >130 mmHg		0.842	Time of ventilation
			SBP >130 mmHg		0.666	Drainage output 24h after surgery
**McIlroy *et al***.# [45]	2019	793	SBP >145 mmHg	0.58 (0.08–4.42)	0.6	Unplanned return to operating room for bleeding on postoperative day 0 or 1
			MAP >95 mmHg	1.32 (0.30–5.81)	0.71	Unplanned return to operating room for bleeding on postoperative day 0 or 1
**Kang *et al***. [32]	2020	1,950	Intraoperative HTN (SBP ≥160 mmHg assessed more than 5 min apart)	1.608 (1.056–2.448)	0.027	Emergence agitation
**McCarthy *et al***. [44]	2020	212	Maximum SBP day 1 (increases of 10 mmHg)	0.85 (0.73–0.98)	0.031	Functional independence
			Maximum SBP day 2 (increases of 10 mmHg)	0.90 (0.84–0.95)	0.042	Functional independence
			Maximum SBP day 3 (increases of 10 mmHg)	0.59 (0.47–0.74)	<0.0001	Functional independence
			Peak SBP change from day 1 to 2	-	-	Functional independence
			Peak SBP change from day 2 to 3	0.97 (0.95–0.99)	0.006	Functional independence
**Morton and Vandal**. [48]	2015	621	Every 10 points rise of highest SBP recordings	1.39 (1.09–1.8)		Hematoma
**Pérez *et al***. [52]	2020	282	Post craniotomy severe HTN (SBP ≥170 mmHg)	10.80% Incidence	0.017	Renal failure
**Post *et al***. [53]	2012	285	SBP ≥150 mmHg	Incidence	0.78	Anastomotic leakage
			DBP ≥90 mmHg	Incidence	0.008	Anastomotic leakage
**Zheng *et al***. [76]	2020	173	24h post-CAS SBP <120 mmHg	1.70% Incidence	0.002	Unfavourable discharge
			24h post-CAS SBP 120–130 mmHg	0% Incidence		Unfavourable discharge
			24h post-CAS SBP >130 mmHg	22.20% Incidence		Unfavourable discharge

Abbreviations: CAS: carotid angioplasty stenting, DBP: diastolic blood pressure, HTN: hypertension, ICU: intensive care unit, LoS: length of stay, MAP: mean arterial pressure, min: minutes, SBP: systolic blood pressure.

^#^ These articles refer to cardiac surgery.

*Generally, OR (95%CI), unless otherwise stated.

#### Blood pressure variation and control of mean arterial pressure

In non-cardiac surgeries, variation in BP (Table 13) has been reported to be correlated with adverse outcomes, such as primary graft non-function, ICU ventilator for more than 3 days, sepsis, prothrombin (PT) >16, poor early graft function and re-transplant, however, no data was available for these outcomes [22]. Additionally, an absolute fractional change of 10% in MAP was described as being protective against primary graft non-function [22]. Moreover, a work studying the influence of control of intraoperative MAP showed that the control of MAP between the range 80–95 mmHg had a protective action against hospital-acquired pneumonia, admission to the ICU and length of stay in ICU [72]. Lastly, the study by Zevallos *et al*. [75] analyzed the mean differences between intraprocedural SBP, DBP, and MAP, comparing cases and controls of patients developing contrast-induced neurotoxicity after neurointerventional procedures, and found statistically significant differences in all of them and concluded that HTN might have a role in increasing blood-brain barrier permeability, allowing the leak of the contrast.

**Table 13:** Diverse outcomes associated with perioperative control of mean arterial pressure and blood pressure variation.

Author	Year	Sample size	Definition of measure	Result	P-value	Event
**DeMaria *et al***. [22]	2013	827	Absolute fractional change in MAP <10%	Protective against adverse outcome (data not shown)		Primary graft non-function
			MAP <50 mmHg	Correlated with adverse event (data not shown)		Primary graft non-function
			MAP <50 mmHg	Correlated with adverse event (data not shown)		ICU ventilator >3 days
			MAP <50 mmHg	Correlated with adverse event (data not shown)		Sepsis
			MAP <50 mmHg	Correlated with adverse event (data not shown)		PT >16
			MAP >120 mmHg	Correlated with adverse event (data not shown)		Poor early graft function
			MAP >120 mmHg	Correlated with adverse event (data not shown)		Re-transplant
**Wu *et al***. [72]	2017	678	MAP 65 to 79 mmHg	3.90% Incidence	0.542	Surgical site infection
			MAP 80 to 95 mmHg	3.40% Incidence		Surgical site infection
			MAP 96 to 110 mmHg	3.40% Incidence		Surgical site infection
			MAP 65 to 79 mmHg	11.30% Incidence	0.014	Hospital acquired pneumonia
			MAP 80 to 95 mmHg	6.70% Incidence		Hospital acquired pneumonia
			MAP 96 to 110 mmHg	10.40% Incidence		Hospital acquired pneumonia
			MAP 65 to 79 mmHg	8.40% Incidence	0.015	Admission to ICU
			MAP 80 to 95 mmHg	4.40% Incidence		Admission to ICU
			MAP 96 to 110 mmHg	7.60% Incidence		Admission to ICU
			MAP 65 to 79 mmHg	2 (1–7) Median (IQR)	0.025	Stay in ICU
			MAP 80 to 95 mmHg	1 (1–3) Median (IQR)		Stay in ICU
			MAP 96 to 110 mmHg	2 (1–6) Median IQR)		Stay in ICU
**Zevallos *et al***. [75]	2020	33	SBP cases—SBP controls	23.41 Average intraprocedural values	0.005	Contrast-induced neurotoxicity
			DBP cases—DBP controls	10.67 Average intraprocedural values	0.008	Contrast-induced neurotoxicity
			MAP cases—MAP controls	13.79 Average intraprocedural values	0.008	Contrast-induced neurotoxicity

Abbreviations: DBP: diastolic blood pressure, CI: confidence interval, ICU: intensive care unit, IQR: interquartile range, MAP: mean arterial pressure, OR: odds ratio, PT: prothrombin, SBP: systolic blood pressure.

## Discussion

The main finding of this work is the existence of evidence supporting the burden of BP excursions in patients undergoing cardiac and non-cardiac surgeries that entail higher risks for negative outcomes after surgery. This review includes a wide range of surgical procedures and addresses several surgical outcomes that could be related to organ damage. There is also evidence that perioperative BP management is important for graft survival, flap loss, and length of hospital stay [18, 22, 33].

What can be highlighted from the results is that it is not just acute HPT or acute HTN that play a role in the development of adverse post-surgical outcomes but also the variation or lability in the values of BP from the patients’ baseline. It is important to characterize each patient’s baseline BP values and assess the intrapatient variability; this approach might have more clinically relevant results than establishing standard thresholds for all the patients [71, 77]. A few studies also incorporated time spent outside of the determined acceptable BP range as a critical factor [3, 46], as adverse outcomes seem to be related to the time the patient spends above or below the optimal ranges: the longer the time, the higher the risk [46, 55, 58, 62, 63, 67]. Regarding long-term outcomes, it seems that no differential long-term effects are expected in the absence of short-term effects, however, further evidence would be needed in order to confirm this hypothesis due to the difficulty of tracing long-term consequences back to the surgery. Nonetheless, a need for the intensification of periopertive medicine has been reported due to the emergence of long-term consequences of postoperative complications; there is an opportunity for the improvement of follow-up, rehabilitation programs, and the fine-tuning of medical management [78].

What has also been seen is that within cardiac surgery all outcomes are significantly associated with BP excursions. Even though this might be expected, there is also evidence for the relationship of HPT and HTN with negative outcomes such as AKI, stroke, discharge to a nursing facility or hospice care, or even death, in non-critically ill trauma surgeries [60] and other type of surgeries.

We highlight the associated risks, especially for severe HPT and HTN [12, 46, 74]. AMI and myocardial injury were also significant events that have been researched extensively, as HPT and HTN have been associated with the risk of both [11, 12, 24, 59, 66, 67]. Both HPT and HTN have also both been described as influencing the risk of stroke in patients undergoing non-cardiac surgeries.

More research needs to be done in order to determine the best strategies to deal with these morbidities in addition to developing new therapeutic strategies to better utilize current medications. Furthermore, a recent meta-analysis assessing the effect of vasoactive drugs (dobutamine, ephedrine, norepinephrine, nitroglycerine, theodrenaline/cafedrine, dopamine, dopexamine, colloids, phenylephrine, inotropes, nitrates, adrenaline) in the perioperative setting has concluded that these drugs might reduce postoperative complications and length of hospital stay in adults having major abdominal surgery [79]. It is clear that the opportunity loss of these agents controlling BP excursions in perioperative settings could be lower or partially compensated by the minimization of health resources that would be required to manage related complications. In this regard a few articles have provided some preliminary insights. Aronson *et al*. analyzed the impact of IV antihypertensive treatment for the management of perioperative BP during cardiac surgery and found that there was an association with shorter time to extubation and shorter ICU stays [80]. For some authors, anesthetic drugs and opioids can be used to control HTN [81–83]. However, their use can imply an increased risk of HPT and for this reason IV BP lowering drugs ideally should be used to control acute HTN episodes [59, 67, 84]. From a pharmacodynamic perspective, it would be reasonable to opt for a strategy that uses a specific type of therapy for each desired pharmacological effect for comprehensive intraprocedural patient management, to optimize their perioperative clinical evolution. Therefore, an opioid (e.g., remifentanil) would be the best option for managing acute pain, while keeping the level of anesthesia achieved separated from pain relief with the use of a hypnotic agent (e.g., propofol) and resorting to an IV antihypertensive that allows rapid, controlled, and predictable reductions in BP within a desired range. This evidence supports the view that new anti-hypertensive and vasoactive agents with favorable pharmacodynamic and pharmacokinetic properties might have a role in many clinical areas and thus, it would be pertinent to further investigate their role to guarantee the fine-tuning of BP and an individualized therapy for patients.

For the management of acute HPT there are also many therapeutic alternatives as described in a recent publication [85]. Fluid administration is a non-pharmacological approach intended to increase stroke volume and cardiac output but other options are also available within the pharmacological armamentarium, such as vasopressors and positive inotropic agents including ephedrine, phenylephrine; additional options are vasopressin and terlipressin, norepinephrine, epinephrine, and other less used agents, angiotensin II, vitamin C, hydroxicobalamin, dopamine, dobutamine, or milrinone. Recommended pharmacological treatments in case of acute HPT in the operating room are epinephrine or phenylephrine in case of mild HPT, and epinephrine in case of significant or refractory HPT [86].

Another study by Aronson *et al*. examined BP variability and demonstrated that by controlling BP during cardiac surgery, patients spent less time on mechanical ventilation and in the hospital, therefore avoiding the associated adverse outcomes and economic costs [5]. Getsios *et al*. took an in-depth look at clevidipine and found that it provided higher cost savings than other IV antihypertensives [4]. Keuffel *et al*. used Monte Carlo simulations to determine the association between intraoperative HPT and hospital expenditures, and concluded that limiting HPT during non-cardiac surgery will have an impact on hospital cost reductions, which may be highly relevant for resource allocation decisions [3]. This literature research also provides a basis for further research and potential economic models of reducing complications related to HPT and HTN.

### Limitations

Although the methodology applied in this research meets international guidelines in the field, a few limitations should be noted. Firstly, in the absence of international guidelines on perioperative HPT and HTN definition (BP thresholds), articles vary greatly in how they define HTN and HPT. This could lead to issues when trying to compare results across studies and prevents performing a meta-analysis in order to assess the effect based on the published evidence due to the heterogeneity among studies. Also, there may be a publication bias that impedes the dissemination of negative findings.

Secondly, these sixty-six articles contain a wealth of evidence on the burden of acute HPT and HTN; however, the evidence remains uneven and difficult to synthesize due to differences in statistical presentation and target population.

Finally, any assessment of the burden is also complicated by the fact that the frequency of the long-term outcomes of acute HPT and HTN may be consequences of their short-term effects (e.g., stroke, AKI, hemorrhage, atrial fibrillation, and others.).

## Conclusion

This review presents the available evidence on the burden of acute HPT and HTN across perioperative settings, and what seems to be clear is that HPT, HTN, and even fluctuations in BP from the patient’s baseline, entail a high burden for patients undergoing diverse types of surgeries. This suggests the potential benefit from improved management of BP in terms of short- and long-term effects of surgical procedures in patient outcomes.

## Supporting information

S1 Checklist PRISMA checklist [87]. (DOC)

S1 File Search strategies. (TXT)

S1 Table Quality assessment [10]. (DOCX)
