# Supplementary material for: The burden of perioperative hypertension/hypotension: A systematic review
Source: PLoS One. 2022 Feb 9;17(2):e0263737. doi: 10.1371/journal.pone.0263737 (PMC8827488; doi:10.1371/journal.pone.0263737)
Supplement: S1 Table — (DOCX) [file pone.0263737.s003.docx]

Supplemental Table 3. Studies included in the review (n=66) and their quality assessment results.

| Study title | Author | Year | Population | Sample Size | HPT/HTN | Quality level (SIGN)^#^ | High-Very low |
| --- | --- | --- | --- | --- | --- | --- | --- |
| Association between preoperative pulse pressure and perioperative myocardial injury: an international observational cohort study of patients undergoing non-cardiac surgery [54] | Abbott *et al.* | 2017 | Non-cardiac surgery | 15,057 | HTN | 2++ | Moderate |
| A prospective international multicentre cohort study of intraoperative heart rate and systolic blood pressure and myocardial injury after noncardiac surgery: results of the VISION study [20] | Abbott *et al.* | 2018 | Non-cardiac surgery | 16,079 | HPT/HTN | 2++ | Moderate |
| Associations of intraoperative radial arterial systolic, diastolic, mean, and pulse pressures with myocardial and acute kidney injury after noncardiac surgery [13] | Ahuja *et al.* | 2020 | Non-cardiac surgery | 164,514 | HPT | 2+ | Moderate |
| Modifiable, postoperative risk factors for delayed discharge following total knee arthroplasty: the influence of hypotension and opioid use [14] | Anastasio *et al.* | 2020 | Total knee arthroplasty | 1,033 | HPT | 2+ | Moderate |
| Intraoperative systolic blood pressure variability predicts 30-day mortality in aortocoronary bypass surgery patients [19] | Aronson *et al.* | 2010 | Aortocoronary bypass graft surgery | 7,504 | HPT/HTN | 2++ | Moderate |
| Does perioperative systolic blood pressure variability predict mortality after cardiac surgery? an exploratory analysis of the ECLIPSE trials [28] | Aronson *et al.* | 2011 | Cardiac surgery | 1,512 | BP variability | 2+ | Low |
| Association between intraoperative low blood pressure and development of surgical site infection after colorectal surgery a retrospective cohort study [64] | Babazade *et al.* | 2016 | Colorectal surgery | 2,521 | HPT | 2- | Low |
| High postoperative blood pressure after cardiac surgery is associated with acute kidney injury and death [24] | Balzer *et al.* | 2016 | Cardiac surgery | 5,225 | HTN | 2- | Low |
| Hypotension during hip fracture surgery and postoperative morbidity [19] | Beecham *et al.* | 2020 | Hip fracture surgery | 52 | HPT | 2- | Low |
| Intraoperative hypotension and perioperative ischemic stroke after general surgery: a nested case-control study [39] | Bijker *et al.* | 2012 | Non-cardiac and non-neurosurgery | 48,241 | HPT | 2++ | Moderate |
| Can routine perioperative haemodynamic parameters predict postoperative morbidity after major surgery? [21] | Bonnet *et al.* | 2020 | Non-cardiac surgery | 50 | HPT | 2+ | Moderate |
| Association of intraoperative blood pressure instability with adverse outcomes after liver transplantation [57] | De Maria *et al.* | 2013 | Orthotopic liver transplantation | 827 | HTN | 2- | Low |
| Intraoperative hypotension is associated with adverse clinical outcomes after noncardiac surgery [23] | Gregory et al | 2020 | Noncardiac surgery | 368,222 | HPT | 2++ | Moderate |
| Intraoperative hypotension is associated with myocardial damage in noncardiac surgery: An observational study [60] | Hallqvist *et al.* | 2016 | Non-cardiac surgery | 300 | HPT | 2+ | Moderate |
| Intraoperative hypotension is associated with acute kidney injury in noncardiac surgery: An observational study[48] | Hallqvist *et al.* | 2018 | Non-cardiac surgery | 470 | HPT | 2+ | Moderate |
| Impact of intraoperative hypotension and blood pressure fluctuations on early postoperative delirium after non-cardiac surgery [41] | Hirsch *et al.* | 2015 | Non-cardiac surgery | 594 | HPT | 2++ | Moderate |
| Perioperative optimal blood pressure as determined by ultrasound tagged near infrared spectroscopy and its association with postoperative acute kidney injury in cardiac surgery patients [33] | Hori *et al.* | 2016a | Cardiac surgery | 110 | HPT | 2+ | Low |
| Blood pressure deviations from optimal mean arterial pressure during cardiac surgery measured with a novel monitor of cerebral blood flow and risk for perioperative delirium: a pilot study [42] | Hori *et al.* | 2016b | Cardiac surgery | 110 | HPT/HTN | 2+ | Low |
| The association between mild intraoperative hypotension and stroke in general surgery patients [40] | Hsieh *et al.* | 2016 | Non-cardiac, non-neurosurgery, and non-carotid surgery | 106,337 | HPT | 2++ | Moderate |
| Intraoperative hypotension is a risk factor for postoperative acute kidney injury after femoral neck fracture surgery: a retrospective study [65] | Jang *et al.* | 2019 | Femoral neck fracture surgery | 248 | HPT | 2- | Low |
| Blood pressure coefficient of variation and its association with cardiac surgical outcomes [23] | Jinadasa *et al.* | 2018 | Cardiac surgery | 3,687 | HTN | 2- | Low |
| Risk factors for emergence agitation in adults undergoing thoracoscopic lung surgery: a case-control study of 1,950 patients [32] | Kang *et al.* | 2020 | Thoracoscopic lung surgery | 1,950 | HPT/HTN | 2+ | Moderate |
| Intraoperative hypotension and flap loss in free tissue transfer surgery of the head and neck [51] | Kass *et al.* | 2018 | Head and neck surgery | 445 | HPT | 2- | Low |
| Intraoperative hypotension is not associated with postoperative cognitive dysfunction in elderly patients undergoing general anesthesia for surgery: results of a randomized controlled pilot trial [45] | Langer *et al.* | 2019 | Non-cardiac surgery | 101 | HPT | 1- | Moderate-High |
| Intraoperative arterial blood pressure lability is associated with improved 30-day survival [26] | Levin *et al.* | 2015 | Surgery | 52,919 | HTN | 2- | Low |
| Perioperative risk factors associated with acute kidney injury in patients after brain tumor resection [36] | Li *et al.* | 2020 | Brain tumor resection | 460 | HPT | 2+ | Moderate |
| High variance of intraoperative blood pressure predicts early cerebral infarction after revascularization surgery in patients with Moyamoya disease [37] | Li *et al.* | 2020 | Revascularization surgery (Moyamoya disease) | 1,497 | HPT | 2+ | Moderate |
| Association of intraoperative hypotension with acute kidney injury after liver resection surgery: an observational cohort study [38] | Liao *et al.* | 2020 | Liver resection | 796 | HPT | 2+ | Moderate |
| Postoperative hypotension after noncardiac surgery and the association with myocardial injury [39] | Liem *et al.* | 2020 | Noncardiac surgery | 1,710 | HPT | 2+ | Moderate |
| Perioperative cardiac complications in patients over 80 years of age with coronary artery disease undergoing noncardiac surgery: the incidence and risk factors [40] | Liu *et al.* | 2020 | Non-cardiac surgery | 547 | HPT | 2+ | Moderate |
| Association between perioperative hypotension and delirium in postoperative critically ill patients: a retrospective cohort analysis [44] | Maheshwari *et al.* | 2019 | Non-cardiac surgery | 1083 | HPT | 2++ | Moderate |
| Intraoperative mean arterial pressure variability and 30-day mortality in patients having noncardiac surgery [30] | Mascha *et al.* | 2015 | Non-cardiac surgery | 104,401 | MAP Variability | 2++ | Moderate |
| Preoperative risk and the association between hypotension and postoperative acute kidney injury [43] | Mathis *et al.* | 2020 | Non-cardiac surgery | 138,021 | HPT | 2+ | Moderate |
| Prolonged heightened blood pressure following mechanical thrombectomy for acute stroke is associated with worse outcomes [44] | McCarthy *et al.* | 2020 | Mechanical thrombectomy | 212 | HTN | 2- | Low |
| Association of postoperative blood pressure and bleeding after cardiac surgery [25] | McIlroy *et al.* | 2019 | Cardiac surgery | 793 | HTN | 2++ | Moderate |
| Relationship between intraoperative hypotension and acute kidney injury after living donor liver transplantation: a retrospective analysis [34] | Mizota *et al.* | 2017 | Liver transplantation | 231 | HPT | 2- | Low |
| Association between intraoperative hypotension and hypertension and 30-day postoperative mortality in noncardiac surgery [21] | Monk *et al.* | 2015 | Non-cardiac surgery | 18,756 | HPT/HTN | 2- | Low |
| Postoperative systolic blood pressure as a risk factor for haematoma following thyroid surgery [55] | Morton and Vandal. | 2015 | Thyroid surgery | 621 | HTN | 2- | Low |
| Defining an intraoperative hypotension threshold in association with de novo renal replacement therapy after cardiac surgery [49] | Ngu *et al.* | 2020 | Cardiac surgery | 6,523 | HPT | 2+ | Moderate |
| Blood pressure excursions below the cerebral autoregulation threshold during cardiac surgery are associated with acute kidney injury [38] | Ono *et al.* | 2013 | Cardiac surgery | 348 | MAP below limit of autoregulation | 2++ | Moderate |
| Duration and magnitude of blood pressure below cerebral autoregulation threshold during cardiopulmonary bypass is associated with major morbidity and operative mortality [29] | Ono *et al.* | 2014 | Cardiac surgery | 450 | MAP below limit of autoregulation | 2+ | Moderate |
| Elevated blood pressure after craniotomy: A prospective observational study [52] | Perez et al. | 2020 | Craniotomy | 282 | HTN | 2++ | Moderate |
| Intraoperative blood pressure changes as a risk factor for anastomotic leakage in colorectal surgery [56] | Post *et al.* | 2012 | Colorectal surgery | 285 | HTN | 2+ | Moderate |
| Impact of intraoperative hypotension during cardiopulmonary bypass on acute kidney injury after coronary artery bypass grafting [66] | Rettig *et al.* | 2017 | Cardiac surgery | 1,891 | HPT | 2- | Low |
| The impact of preoperative risk on the association between hypotension and mortality after cardiac surgery: an observational study | Ristovic *et al.* | 2020 | Cardiac surgery | 6,627 | HPT | 2+ | Moderate |
| Relationship between perioperative hypotension and perioperative cardiovascular events in patients with coronary artery disease undergoing major noncardiac surgery [49] | Roshanov *et al.* | 2019 | Non-cardiac surgery | 955 | HPT | 2- | Low |
| Intra-operative hypotension is a risk factor for post-operative silent brain ischaemia in patients with pre-operative hypertension undergoing carotid endarterectomy [56] | Rots *et al.* | 2020 | Carotid endarterectomy | 55 | HPT/HTN | 2- | Low |
| Relationship between intraoperative hypotension, defined by either reduction from baseline or absolute thresholds, and acute kidney and myocardial injury after noncardiac surgery [35] | Salmasi *et al.* | 2017 | Non-cardiac surgery | 57,315 | HPT | 2- | Low |
| Period-dependent associations between hypotension during and for four days after noncardiac surgery and a composite of myocardial infarction and death [14] | Sessler *et al.* | 2018 | Non-cardiac surgery | 9,765 | HPT | 2++ | Moderate |
| Perioperative hypotension and discharge outcomes in non-critically injured trauma patients, a single centre retrospective cohort study [52] | Sheffy *et al.* | 2017 | Trauma surgery | 1,744 | HPT | 2- | Low |
| Intraoperative hypotension, new onset atrial fibrillation, and adverse outcome after carotid endarterectomy [47] | Sposato *et al.* | 2011 | Carotid endarterectomy | 186 | HPT | 2++ | Moderate |
| Association of intraoperative hypotension with acute kidney injury after elective noncardiac surgery [36] | Sun *et al.* | 2015 | Non-cardiac surgery | 5,127 | HPT | 2- | Low |
| Association of intraoperative hypotension with acute kidney injury after noncardiac surgery in patients younger than 60 years old [37] | Tang *et al.* | 2019 | Non-cardiac surgery | 4,952 | HPT | 2- | Low |
| Impact of intraoperative hypotension on hospital stay in major abdominal surgery [53] | Tassoudis *et al.* | 2011 | Abdominal surgery | 100 | HPT | 2++ | Moderate |
| Association between postoperative mean arterial blood pressure and myocardial injury after noncardiac surgery [22] | van Lier *et al.* | 2018 | Non-cardiac surgery | 2,211 | HPT | 2- | Low |
| Association between intraoperative hypotension and myocardial injury after vascular surgery [59] | van Waes. | 2016 | Non-cardiac surgery | 890 | HPT | 2++ | Moderate |
| relationship between intraoperative mean arterial pressure and clinical outcomes after noncardiac surgery: toward an empirical definition of hypotension [13] | Walsh *et al.* | 2013 | Non-cardiac surgery | 33,330 | HPT | 2- | Low |
| Association between intraoperative blood pressure and postoperative delirium in elderly hip fracture patients [46] | Wang *et al.* | 2015 | Hip fracture surgery | 103 | HTN | 2++ | Moderate |
| Intraoperative hypotension and delirium after on-pump cardiac surgery [43] | Wesselink *et al.* | 2015 | Cardiac surgery | 743 | HPT | 2++ | Moderate |
| Concurrence of intraoperative hypotension, low minimum alveolar concentration, and low bispectral index is associated with postoperative death [67] | Willingham *et al.* | 2015 | Any surgery | 13,198 | HPT | 2- | Low |
| Intraoperative blood pressure variability predicts postoperative mortality in non-cardiac surgery-a prospective observational cohort study [31] | Wiorek and Krzych. | 2019 | Non-cardiac surgery | 835 | BP Variability | 2++ | Moderate |
| Optimal blood pressure decreases acute kidney injury after gastrointestinal surgery in elderly hypertensive patients: a randomized study optimal blood pressure reduces acute kidney injury [32] | Wu *et al.* | 2017 | Gastrointestinal surgery | 678 | Control of MAP | 2++ | Moderate |
| Postoperative hypotension and surgical site infections after colorectal surgery: a retrospective cohort study [50] | Yilmaz *et al.* | 2018 | Colorectal surgery | 5,896 | HPT | 2- | Low |
| Association of perioperative blood pressure with long-term survival in rectal cancer patients [27] | Yu *et al.* | 2016 | Colorectal surgery | 358 | HTN | 2++ | Moderate |
| Greater intraprocedural systolic blood pressure and blood pressure variability are associated with contrast-induced neurotoxicity after neurointerventional procedures [74] | Zevallos *et al.* | 2020 | Neurointerventions | 33 | BP Variability | 2++ | Moderate |
| Perioperative blood pressure control in carotid artery stenosis patients with carotid angioplasty stenting: a retrospective analysis of 173 cases [75] | Zheng *et al.* | 2020 | Carotid angioplasty stenting | 173 | HTN | 2- | Low |

Abbreviations: BP, blood pressure; HPT, hypotension; HTN, hypertension; MAP, mean arterial pressure.
^#^Quality level (from higher to lower): 1++, 1+, 1-, 2++, 2+, 2-, 3, 4. High (1++, 1+), Moderate-High (1-), Moderate (2++), Low (2+, 2-), Very low (3, 4)
